# Strategies to Mitigate and Treat Orthopaedic Device-Associated Infections

**DOI:** 10.3390/antibiotics11121822

**Published:** 2022-12-15

**Authors:** Darragh G. Kennedy, Aoife M. O’Mahony, Eamonn P. Culligan, Caitriona M. O’Driscoll, Katie B. Ryan

**Affiliations:** 1 School of Pharmacy, University College Cork, T12 K8AF Cork, Ireland; 2 Department of Biomedical Engineering, Columbia University, New York, NY 10027, USA; 3 Department of Biological Sciences, Munster Technological University, T12 P928 Cork, Ireland

**Keywords:** orthopaedic implants, infection, biofilm, bioinspired, drug delivery, implant coating, polymer, antimicrobial, nanotechnology, medical device

## Abstract

Orthopaedic device implants play a crucial role in restoring functionality to patients suffering from debilitating musculoskeletal diseases or to those who have experienced traumatic injury. However, the surgical implantation of these devices carries a risk of infection, which represents a significant burden for patients and healthcare providers. This review delineates the pathogenesis of orthopaedic implant infections and the challenges that arise due to biofilm formation and the implications for treatment. It focuses on research advancements in the development of next-generation orthopaedic medical devices to mitigate against implant-related infections. Key considerations impacting the development of devices, which must often perform multiple biological and mechanical roles, are delineated. We review technologies designed to exert spatial and temporal control over antimicrobial presentation and the use of antimicrobial surfaces with intrinsic antibacterial activity. A range of measures to control bio-interfacial interactions including approaches that modify implant surface chemistry or topography to reduce the capacity of bacteria to colonise the surface, form biofilms and cause infections at the device interface and surrounding tissues are also reviewed.

## 1. Introduction

Orthopaedic medical devices are a significant area of focus in healthcare provision. They are primarily used for fixation of long bone fractures and non-unions, spinal fracture stabilisation and replacement of diseased (arthritic) or damaged joints [1]. Intramedullary nail fixation devices have been used in the treatment of closed fractures of the long bones (femur and tibia), whilst hip and knee arthroplasties involve replacement of joints by implantation of prosthetic devices. These procedures are common, with an estimated six hundred thousand joint prostheses and two million fracture fixation devices implanted in the United States alone [2]. In the case of orthopaedics, metallic implants are primarily employed as they provide the necessary mechanical strength in high load-bearing environments that are subject to repeated cyclic loads and strains [3]. The success in restoring biological function and implant integration with host tissue (where desirable) can be hampered by many variables including patient factors, procedural complications and those owing to the implant device itself (stress-shielding effects). The leading causes of implant failure are attributed to aseptic loosening and infection [4].

All surgical procedures are associated with a risk of infection, the incidence varying from 0.5% to 4% in total hip and knee implants, respectively, even with the systemic administration of antibiotics prior to surgery [5]. However, more substantive infection rates (up to 40%) may occur in cases of revision surgeries on failed prosthetic joints [6], whilst up to 44% of open fractures are reported to develop post-operative infections [2]. The health and economic impacts of orthopaedic device-related infection (ODI) to patients and healthcare providers are significant, owing to longer hospital stays and greater hospital costs for revision surgeries where infection is implicated compared to aseptic cases [7]. A review of the Nationwide Inpatient Sample (Q4 2005–2010) to quantify the clinical and economic burden of periprosthetic infection revealed infection to be the leading indication for revision total knee arthroplasty procedures and the third most common reason for revision total hip arthroplasties. These patient cohorts had the longest hospital stays and with the exception of periprosthetic fracture patients, the highest costs [8]. Together with the expected increase in orthopaedic medical device usage (e.g., a sixfold increase in arthroplasty procedures by 2030 has been projected) [9], it is clear that ODIs represent a major concern for patients and healthcare providers.

Drug treatment regiments typically comprise part of the management and treatment of device-related infections. Gentamicin is the preferred antimicrobial agent used in orthopaedic device infections [10]. However, challenges associated with polymicrobial infections, drug resistance and limitations of systemic drug delivery including subtherapeutic drug concentrations at the target site due to compromised vascularity and poor perfusion can lead to treatment failure and poor patient outcomes. Depending on the severity of the infection, previous attempts to cure the infection, antibiotic resistance, symptoms present and their duration, patient risk factors, comorbidity and implant stability [11,12], intervention may involve surgical debridement with retention of the prosthesis, one- or two-stage implant exchange or implant removal without replacement. In very serious cases of uncontrolled infection, amputation may be necessary [12,13]. Local delivery of antibiotics has already been successfully translated to the clinic in the guise of antibiotic-loaded bone cements (ALBC), antibiotic-impregnated collagen sponges and poly (methyl methacrylate) (PMMA) beads [14]. Antibiotic-impregnated PMMA cements have been employed as spacers in two-stage revision surgeries or the management of infections in revision procedures [15]. Local antibiotic elution for prophylactic purposes has been employed more readily in joint replacement surgery in comparison to fracture treatment as the prosthetic joints are stabilized using ALBC [16]. These approaches have a number of drawbacks including incomplete and variable drug release, the non-degradable properties of PMMA and the incompatibility of some drugs with PMMA arising from exothermic reactions during curing [12]. Additionally, the presence of antibiotics may decrease the internal strength of the cement, thereby decreasing its overall mechanical performance [17,18].

Consequently, the problem of implant failure owing to infection has led researchers to examine implant design in a bid to address this problem. This review will focus on the progress to date in the development of the next-generation orthopaedic medical devices to control ODI. Strategies to mitigate against the risk of infection including the application of surface coatings will be discussed. Bactericidal surfaces that inactivate bacteria primarily by chemical mechanisms using antimicrobial agents or materials with intrinsic antibacterial activity will be discussed. A range of measures to control bio-interfacial interactions including approaches that modify implant surface chemistry or topography to reduce the capacity of bacteria to colonize the surface, form biofilms and cause infections at the device interface and surrounding tissues will also be reviewed.

## 2. Orthopaedic Device-Related Infection (ODI)

### 2.1. Origin and Causative Organisms

Infection may occur at the perioperative stage (sources of infection include surgical equipment, clothing worn by operating theatre personnel and the atmosphere of the operating room) [19], contiguously (wound contamination occurs during trauma—in penetrating injuries or from a source adjacent to the wound) or the infection may be hematogenous in origin (infection spread by the lymph or blood occurs from a distant site of infection) [20]. The primary causative organisms in orthopaedic infections are *Staphylococcal* in origin [21,22] with *Staphylococcus aureus* (*S. aureus*) and coagulase negative staphylococcus species a factor in more than 50% of prosthetic hip and knee infections [23]. Although other Gram-positive (*Streptococcus*, *Enterococcus*, *Propionibacterium* species) and Gram-negative (*Pseudomonas aeruginosa, Escherichia coli*) pathogens are also implicated [24,25,26]. Epidemiological studies confirm the predominance of staphylococci and signal the complexity of the treatment challenges due to the broad spectrum, variability and virulence of pathogens implicated, changing species profiles in early and late infections, and the consistent presence of polymicrobial infections [24,27,28,29]. The development of methicillin resistant *S. aureus* (MRSA) strains further complicates the problem. Conventional culture tests are used to diagnose infections; however, they are limited by low sensitivity in detecting the causative organisms. Advancements in diagnostic technology offering enhanced sensitivity, e.g., next-generation sequencing, will likely lead to more comprehensive analysis and detailed insights into the infectious organisms in patients’ samples [30].

A further complication includes the presence of infections with a biofilm aetiology, where microbes form multicellular communities on biological and non-biological surfaces [31]. Biofilms help retain nutrients and offer protection against the patient’s immune system [32]. The presence of foreign bodies, e.g., implants are thought to further exacerbate the infection risk [33], as they provide substrates for colonisation and biofilm formation. Studies have shown that the presence of an implant influenced *S. aureus* infection pathogenesis in a subcutaneous guinea pig model with 10^2^ colony-forming units (CFU) causing infection in the majority of animals, whereas in the absence of the foreign body no infection was present even at 10^8^ CFU [34]. There may be further complications if the presence of the implant evokes a foreign body response attributed to “non-self” materials, compromising the immune system and leading to an increased infection risk [35].

Infections have been classified based on their occurrence post-operatively. “Early” infections develop less than 3 months after surgery, those categorized as “delayed” occur within a 3–24 month timeframe and “late” manifest greater than 24 months after surgery [20]. Infection with virulent organisms, e.g., *S. aureus* and Gram-negative bacilli at the time of implantation typically gives rise to acute infections within the first 3 months post-operatively, while infections with less virulent organisms are more typically associated with chronic infections over time frames of many months or years after surgical implantation [13]. The symptoms of early infections typically manifest as pain, swelling, erythema and fever. Delayed infections are characterized by persistent joint pain and implant loosening that may be difficult to differentiate from aseptic loosening [36]. Indeed, infection is the primary cause of failure following total knee arthroplasty [11]. When orthopaedic-associated infection goes untreated, it can progress to osteomyelitis and bone destruction [37].

### 2.2. Biofilm-Related Infections

#### 2.2.1. Biofilm Formation

The presence of a biofilm aetiology in device-related infections is widely recognized as a primary pathogenic mechanism contributing to infections that are persistent and difficult to eradicate [38]. Biofilms have been described by Donlan and Costerton (2002) as “microbially derived sessile community characterized by cells that are irreversibly attached to a substratum or interface or to each other, are embedded in a matrix of extracellular polymeric substances that they have produced, and exhibit an altered phenotype with respect to growth rate and gene transcription” [39]. Studies of biofilms have revealed differentiated, structured groups of cells with community properties that are responsive to the stresses of their surrounding environment [40].

**Figure 1:**
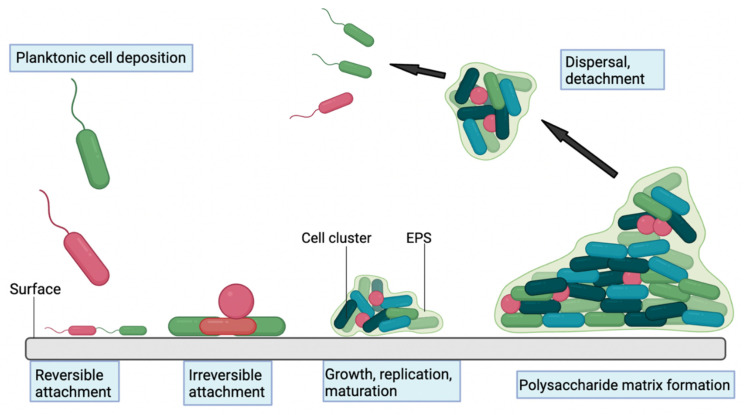
Schematic representation of the main steps in biofilm formation on a surface. Biofilm formation is a cyclic process that begins with surface contact by single planktonic cells. Cells go through reversible attachment whereby bacteria attach to a surface via their flagella or cell pole, to irreversible attachment when flagella reversal rates decrease, and biofilm matrix component production occurs. Biofilm maturation follows, and dispersion occurs due to extracellular polymeric substance (EPS) degradation. Created with BioRender.com.

The primary conceptual framework for biofilm formation involves four key stages implicit in biofilm development beginning with initial attachment of planktonic bacteria on the surface, followed by proliferation and cell accumulation, biofilm maturation and cell detachment [29] (Figure 1). Bacterial adhesion on material surfaces proceeds by a two-step process. Attachment in the initial, reversible physical phase is mediated by non-specific factors including implant surface properties (e.g., hydrophobicity, surface tension, electrostatic interactions), whereas molecular and cellular interactions between the bacteria and implant substrate dominate in the second, irreversible phase [31,41]. Additionally, the host inflammatory response to the implant can play an important role in facilitating bacterial adhesion to the surface [31]. The material substrate and bacteria can influence the adhesion process in different ways. Adsorption of host proteins including fibronectin and fibrinogen on the implant surface after exposure to physiological fluids can provide anchorage sites for *S. aureus* cells, mediated by bacterial surface adhesion proteins, e.g., MSCRAMMs (microbial surface components recognising adhesive matrix molecules) [31]. The MSCRAMMs are complex receptor proteins anchored to the bacterial cell and thought to be multifunctional with some MSCRAMMS recognising extracellular matrix (ECM) ligands, acting as invasins or facilitating bacterial cell accumulation [42]. Cells form microcolonies on the surface, which arises due to translocation across the surface or due to clonal growth of attached cells. The discrete cell clusters grow in size to form macrocolonies with a classical mushroom-like tower structure interspersed with fluid-filled voids proposed to support the diffusion of nutrients and waste by-products [43]. Other mechanisms implicated in biofilm maturation include the release of eDNA and the production of an extracellular polysaccharide matrix [44]. In staphylococci, the presence of polysaccharide intercellular adhesin (PIA) in the polymeric matrix is an important factor in bacterial adhesion and accumulation within the biofilm [45]. Other protein factors have also been identified to play a role in staphylococcal biofilm formation including the accumulation associated protein (Aap) in *Staphylococcus epidermidis* (*S. epidermidis*) isolated from human infections [46].

Biofilm production can be impacted by environmental conditions and changes in cell density through the quorum-sensing system, whereby cell-to-cell communication between bacteria mediated through the release of signalling molecules can coordinate changing patterns of gene expression within bacterial populations [31,47]. The continued expansion of bacterial colonies creates an increase in the internal pressure in the biofilm that is followed by detachment of cells and reversion to the planktonic state, granting them the ability to infect surrounding tissue or initiate biofilm development elsewhere [48]. Dispersal of clumps of cells encased in extracellular polymer substances more reminiscent of attached biofilm rather than the planktonic state is also a feature of *S. aureus* biofilms [49]. Other factors expressed under the control of quorum sensing are also implicated in biofilm disruption; detachment and bacterial dispersion include the production of β-type phenol-soluble modulins (a group of pro-inflammatory peptides with surfactant-like properties), extracellular proteases and murein hydrolases [29,50,51].

#### 2.2.2. Biofilms—The Clinical Consequences

Bacterial growth in aggregates and encased in the biofilm “slime” matrix affords the microbes protection from environmental (shear forces) and the host’s defences (opsonisation and phagocytosis) [39,49]. Sessile bacteria cells are also less susceptible to antibiotics compared to non-attached, planktonic cells. Biofilm bacteria are reported to tolerate exposure to drug concentrations ranging from 10 to 1000 times that required to eliminate genetically equivalent planktonic forms [52]. This reduced susceptibility has also been attributed to poor antimicrobial penetration of the biofilm due to impaired drug diffusion or the retardant effect of the polymeric matrix [53,54]. Reductions in bacterial metabolic activity can lead to profound differences in antibiotic susceptibility [55]. Indeed, the heterogeneity of a biofilm matrix may represent an important survival strategy because differences in the metabolic activity of the cells increase the likelihood that some bacterial cells may survive a metabolically directed attack [56]. This reduced antibacterial susceptibility has important clinical implications as it necessitates high doses of antibiotics to target and eradicate infection, often with limited success. Indeed, infections recalcitrant to antibiotic therapy despite planktonic sensitivity to the drug is one of several clinical and microbiological criteria that have been defined in an attempt to classify and diagnose infections with a biofilm aetiology [49,57,58]. Given the reduced susceptibility to antibiotics, the high proportion of biofilm-based infections (up to 65%) [59], the propensity for these infections to be chronic [57], the difficulty in targeting effective concentrations to the infection using systemic drug delivery and owing to side-effects associated with high-dose antibiotic regimes [60], the most effective strategy to eradicate problematical biofilm infections involves removal of the implanted device and debridement of the surrounding tissue [21]. Such procedures are not always possible and carry the risk of complications, underscoring the challenges biofilm-related infections pose for patients and healthcare providers [61].

## 3. Infection Control and Orthopaedic Implant Design

### 3.1. Orthopaedic Device Materials

A range of materials including metals, ceramics and plastics or a combination of these has been used to construct orthopaedic implants. Orthopaedic implants perform many different functions, which impacts the choice of material used [4]. Strong materials are required for load-bearing applications, while durability and wear resistance are important requirements in articulating surfaces [62]. Key factors influencing material choice include tensile strength, elastic modulus, corrosion resistance, ductility, longevity, potential to release metal debris, and the impact on device functionality and performance [63]. Irrespective of application, desirable features include biocompatibility and a minimal foreign body response. Three generations of orthopaedic biomaterials have been described by Hench and Polak [64]: First generation, bioinert materials, elicit a minimal response from host tissue. Second generation encompass resorbable or bioactive materials that form bonds with the tissue and material, while third generation materials stimulate cell responses to encourage healing.

Metals are commonly used for load-bearing applications in joint replacements, dental implants and fracture-fixation devices in a bid to match desirable mechanical and wear resistance properties with the functional application [65]. Commonly used metals include biomedical grades of stainless steel (e.g., 316L stainless steel), alloys of cobalt including cobalt–chromium–molybdenum (CoCrMo) and titanium (Ti) and titanium-based alloys (Ti-6Al-4V, Ti-6Al-7Nb) [66]. Biodegradable metals including magnesium-based compounds are increasingly investigated as they are biocompatible and mechanically superior to biodegradable polymers [67,68]. However, their strength is lower than other metal substates, and rapid degradation of implants in vivo can lead to the evolution of hydrogen gas that causes inflammation and irritation [67,69].

Second generation, bioactive materials are used in scenarios where device integration with the host tissue and augmentation of healing is required. For example, osteointegration is not a priority in cases where the device is removed following healing (fracture fixation plates, screws). In cases where osteointegration is desirable deposition of ceramic biomaterials including calcium-phosphate-based materials, e.g., hydroxyapatite (HA) and tricalcium phosphate [70,71,72], on implant surfaces is common due to the similarity of these materials to natural bone minerals. Calcium phosphate-based materials with different compositions, chemical and physical properties have been investigated in an effort to control cell adhesion, differentiation behaviour and osteointegration [73]. Other strategies to promote integration of the device include increasing implant surface roughness [74,75] and increasing wettability [76]. However, efforts to support osteointegration can also give rise to increased bacterial attachment and promote the development of infection [77]. Alternative ceramics include alumina, which is exploited for its hardness and wear resistance in arthroplasty to produce femoral head components, while it is combined with zirconia (ZTA) to enhance its toughness [66,78]. Zirconia ceramics are amenable to 3D printing, which advances the prospect to create bespoke, patient-specific devices, thereby addressing problems during implantation due to the mismatch between the patient and prosthesis shape [79].

A range of synthetic polymeric materials are also utilised, including highly cross-linked ultrahigh molecular weight poly(ethylene) (UHMWPE), poly(L-lactic acid) (P_L_LA), polyether–ether–ketone (PEEK) and PMMA [66]. The most well-known is PMMA, a polymeric cement used for hard tissue applications that is inserted between the device and bone to ensure that stress is more uniformly distributed [80]. Antibiotic-loaded cements are also utilised for local antibiotic delivery for infection prophylaxis and treatment [81], and as a spacer in two-stage reconstruction of infected joints [82]. Resorbable P_L_LA-based devices have been used in non-load-bearing applications, e.g., maxillofacial fractures [69]. PEEK, which exhibits many desirable properties, e.g., elastic modulus, strength, wear resistance, radiolucency, and biocompatibility, has been used to manufacture maxillofacial and spinal implants. Drawbacks include its bioinert and hydrophobic properties, and its susceptibility to microbial infections [83].

### 3.2. Infection Control

**Figure 2:**
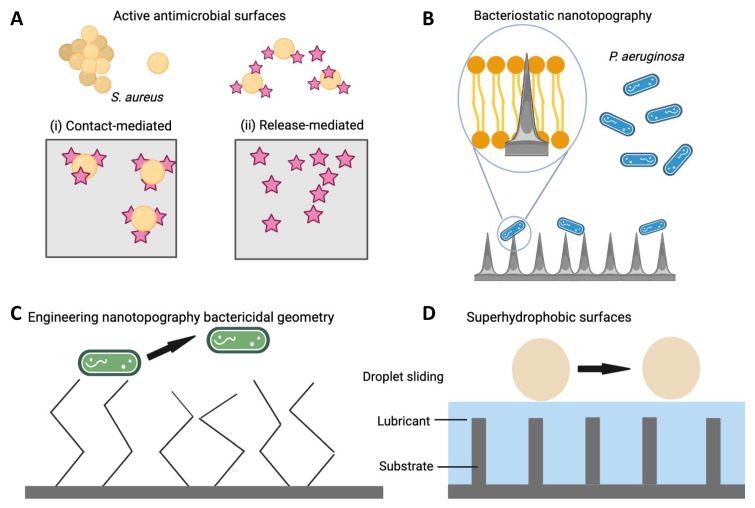
Schematic illustration of various antimicrobial and drug-free strategies currently under investigation to combat device-related infection. (**A**) Active antimicrobial surfaces utilise antimicrobial agents that have been coated on the surface or encapsulated within coatings to kill or impede bacteria growth. They exert their effect when (i) bacteria contact the antimicrobial surface or (ii) when released. Drug-free strategies include engineering (**B**) bactericidal nanotopographies and (**C**) device surfaces to minimise bacterial adhesion including polymeric brushes that impede microbial attachment owing to steric hindrance, or (**D**) the use of superhydrophobic surfaces to repel microbes, such as SLIPS (slippery liquid-infused porous surfaces). Created with BioRender.com.

Extensive research has been undertaken to address the problem of ODI and a range of infection control strategies has been pursued including inhibition of bacterial adhesion, interference with biofilm formation or presentation of antimicrobial agents (Figure 2). Non-eluting coatings that impede or prevent bacterial adhesion through surface chemistry or engineering the surface have been described as passive, while the presentation of antimicrobial agents incorporated on the surface, in a coating or carrier and which prevent infection by killing bacteria are classed as active [1,84]. In the case of implants intended for integration with the host, the competition between microorganisms and host cells to adhere and coat the surface has an important influence on the long-term device success. Gristina and colleagues described this phenomenon as the “Race for the surface” [85]. Repellent surfaces may compromise host integration in the case of permanent implants as many of the mechanisms crucial to host cell adhesion also promote adhesion of microorganisms [86]. This has prompted research in the use of multifunctional coatings that exploit variations in surface chemistry and patterned topography to create both adhesive and non-adhesive sites and have been reviewed elsewhere [86,87,88]. In contrast, the use of coatings that do not promote host integration may be desirable in trauma and fracture fixation, whereby removal of the device after healing may be desirable [89]. The choice of material used to combat biofilm-associated infections has a profound impact on the ensuing foreign body response and should be closely considered [90].

**Figure 3:**
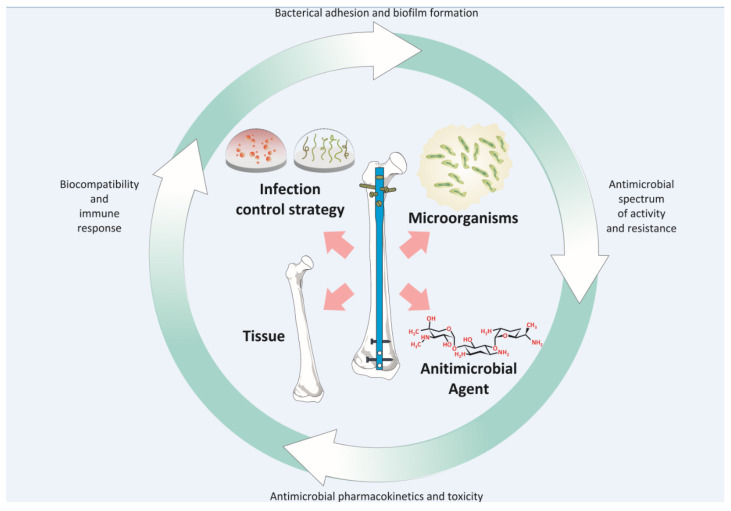
Important considerations impacting the design and clinical performance of orthopaedic devices incorporating antimicrobial agents to control device-related infection.

Table 1 provides an overview of the salient considerations in the design and manufacture of orthopaedic implants for infection control. Irrespective of the infection control strategy, materials employed to tailor the device surface should be biocompatible, possess desirable mechanical properties, enable infection control both in the acute and latent phase and promote integration with the host tissue where desirable [88]. Any added antimicrobial agent should have a spectrum of activity against the primary causative agents. Rational device design for implantation should take account of the interplay (Figure 3) between the key features including the organisms responsible for the infection, their attraction to the implant surface and their susceptibility to the antimicrobial agents [6]. Important prerequisites in device design and infection control strategies include the need to ensure that any advancement does not add complexity to the surgical implantation procedure and that intricate designs do not act as a barrier to scale-up and manufacture [16].

## 4. Antimicrobials and Anti-Biofilm Strategies

### 4.1. Antimicrobial Cargoes and Materials

A diverse array of molecules has been investigated for the purpose of minimising ODI including small drug molecules (e.g., antibiotics), polymers with intrinsic bactericidal activity (e.g., chitosan), ions (silver, zinc, copper), quaternary ammonium salts and antimicrobial peptides (AMPs) [4]. Important considerations when selecting an agent include the stability, tissue compatibility, toxicity, and spectrum of activity. A sizeable body of research has investigated the local presentation of antibiotics (e.g., gentamicin and vancomycin) on the surface of orthopaedic implants to prevent or ameliorate ODI, which has culminated in limited clinical success to date. This may be attributed, in part, to insufficient clinical evidence, concerns over the impact on bone growth, regulatory requirements, cost and controversy surrounding the routine use of antibiotics and the associated risk of resistance. Treatment of orthopaedic infections may be complicated by the diminished or lack of efficacy of some drugs (e.g., gentamicin and vancomycin) against intracellular bacteria prompting investigations based on combinations such as those involving rifampicin, which targets intracellular bacteria [87]. Indeed, the premanufacture of devices coated with a single antimicrobial agent may represent an oversimplified solution to the problem given the challenges of infections with a biofilm aetiology, incidence of polymicrobial infections and epidemiological differences [49]. Efforts to address some of these limitations have focused on the use of carriers or coatings applied at the time of the surgical procedure [91]. Notably, even with diagnostic efforts to culture and identify causative organisms, isolated bacterial strains deemed susceptible to an antibiotic in laboratory testing can be completely resistant to the same antibiotic when growing under a biofilm [92]. Despite the limitations highlighted here, data for local antibiotic delivery using ALBC suggests decreased infection rates in primary procedures [93], or as part of a wider infection control strategy [94], while a recent meta-analysis of in vivo studies concluded that antibiotic coatings on titanium offered a promising infection prevention approach [95].

The rise in antibiotic resistance has prompted intensive research on the development of new antimicrobials, the chemical modification of existing drugs and the development of materials with intrinsic antimicrobial activity. However, despite considerable investment, the pace of new drug development and translation of new antimicrobial drugs to the clinical setting has been slow and lagged the emergence of multi-drug resistant bacterial strains [96,97]. Scientists are also exploring new therapeutic moieties to treat infections, which are at varying stages of development and acceptance by the scientific community. These include metallic ions and nanoparticles (NPs), biological agents including AMPs, phage technology and gene-editing enzymes [98]. Indeed, quorum-sensing inhibitors and quenchers, which impede biofilm formation by targeting the microbial communication mechanisms, have also been investigated. Their role in biofilm formation and/or maturation has been reviewed elsewhere [99] and is discussed in the next section. Multi-drug combinations or the combination of drugs with either antimicrobial materials or quorum-sensing inhibitors have also been investigated to enhance the antibacterial spectrum, create synergy and circumvent the risk of bacterial resistance. More recently, attempts to develop novel antimicrobials for orthopaedic applications investigated bone-binding antibiotic (BBA-1), which was produced via a two-step chemical conjugation of cationic selective antimicrobial-90 (CSA-90), and the bisphosphonate, alendronate [100]. BBA-1 demonstrated rapid binding to bone mineral and showed potent antibacterial activity against *S. aureus* and MRSA in vivo. Data also indicated the potential to ensure lasting activity against osteomyelitis after systemic delivery. Research into the use of natural (e.g., chitin and chitosan) and synthetic polymers with intrinsic antimicrobial activity for infection control for different applications (health, food, packaging) is well established. [101]. Cellulose-based materials with lignin or carboxylic functional groups have been proposed to be useful in anti-biofilm engineering [102]. Antimicrobial materials represent an attractive alternative to antimicrobial drugs and can be used to functionalize surfaces without adversely affecting the antimicrobial properties, although antimicrobial efficacy is dependent on the material and pathogen. Research in this area has focused on chemical modification of the materials to optimize physicochemical properties (e.g., solubility) and enhance antimicrobial functionality or exploiting key structural features to generate new antimicrobial materials [101].

Of the various metal ion-releasing surfaces, silver (Ag) is the most widely investigated to mitigate against the risk of infection. It has been used medically in wound dressings and for the prophylaxis and treatment of burns. Silver application has evolved from the ionic form in silver nitrate, or combined with antibacterial sulphonamide drugs in silver sulfadiazine, towards its use as a coating on urinary catheters or in NP form in dressings [103]. Although its popularity declined with the introduction of antibiotic drugs, there has been a resurgence in interest due to emergence of antibiotic-resistant bacterial strains. In the context of device-associated infection prevention, it has been presented using a number of delivery vectors including Ag-doped ceramic and polymer coatings, Ag NP and thin-films [48,104,105,106], with ion-releasing coatings demonstrating good antibacterial activity in vitro [107,108]. More recently, silver was encapsulated in carboxymethyl cellulose (CMC) microparticles by spray-drying and included in a calcium phosphate bone cement [109]. Loading silver into the microparticles rather than the cement itself yielded faster release of silver within the first few days, yet mitigated the undesirable silver–cement interactions during setting. However, concerns over toxicity in some eukaryotic cell lines when high concentrations are used and the lack of sufficient clinical evidence of Ag efficacy in in vivo orthopaedic applications has limited the widespread use of this approach [16,110]. Several other metal ions have also been investigated for their antimicrobial effects. A copper (Cu)-containing bioactive glass–collagen scaffold composite was demonstrated to promote bone generation owing to its osteogenic and angiogenic potential [111]. Its antimicrobial capacity was demonstrated through elution of Cu ions.

The inclusion of AMPs in device coatings has also been proposed to combat infection. These compounds, described as next-generation antibiotics because they are far less susceptible to the development of pathogen resistance compared to conventional antibiotics, are potent and exhibit rapid and broad-spectrum antibacterial activity [112,113]. AMPs play a key role in natural defence mechanisms and have been isolated from various natural sources including bacteria, fungi, viruses, plants, and animals. They are generally composed of between 12–50 amino acids and exert their action by altering the cell membrane. Four main classes exist, including (i) cationic, (ii) anionic, (iii) anionic and cationic peptides with disulphide bonds and (iv) linear, cationic peptides with an alpha-helical structure [114]. Recently, the anti-biofilm properties of human beta-defensin 2 in *P. aeruginosa* biofilms were reported, including structural changes, outer membrane protein alteration and interference with transfer of biofilm precursors into the extracellular space [115]. Their usage is limited by problems with stability, cost, and the potential for antigenicity. Scientists are using natural peptides as templates to create synthetic compounds that vary in amino acid content, sequence, and chain length to overcome some of the drawbacks and produce enhanced activity and reduced cytotoxicity [116,117,118].

### 4.2. Combatting Bacterial Biofilms

Given that biofilm formation plays a key role in the persistence of infection enabling bacteria to evade the host immune response and resist drug treatment, it too has become an important target in eradicating device-related infection. Methods to reduce biofilm-associated infections have focused on developing polymeric materials to reduce non-specific bacterial adhesion [119] by targeting particular adhesion mechanisms and promoting biofilm disaggregation; these have been reviewed elsewhere [120,121,122]. Materials with intrinsic antifouling properties are attractive due to their ability to prevent biofilm formation. The most well-known materials, poly(ethylene glycol) (PEG) brushes and zwitterionic polymers are discussed in Section 7. High-throughput screening approaches using combinatorial polymer microarrays have proven fruitful for the discovery of novel polymeric materials resistant to bacterial attachment [119,123].

Modulation of bacterial communication by targeting quorum-sensing using quorum-quenchers and quorum-sensing inhibitors (QSI) or indeed activation of the *agr* quorum-sensing system to produce staphylococcal proteases are more novel approaches to disrupting biofilm [38]. Variability in the *agr* system across staphylococcal strains would necessitate an additional diagnostic step in the clinical setting to accurately identify *agr* type prior to treatment with activators such as autoinducing peptides (AIP) because each *agr* type recognizes a different AIP structure [120,124]. Several promising QSI have been tested in vivo. In a model of biofilm formation, antibodies against quorum-sensing molecules (AtlE, the autolysin of *S. epidermidis*, GroEL, a bacterial heat shock protein and polysaccharide intercellular adhesion, essential in cell–cell adhesion in bacteria) reduced biofilm formation in an in vitro model of *S. epidermidis* [125]. Anguita-Alonso et al., (2007) applied an RNAIII-inhibiting peptide (RIP) to bone cement beads, which prevented *S. aureus* infection in vivo in a rat model [126]. Despite this, there is debate surrounding this approach given that exact mechanisms are unclear and application of QSI to inhibit biofilm formation in one species could lead to promotion of biofilm formation in another [38].

Three-dimensional printing of anti-biofilm structures has increased in popularity in recent years. Some monomers are themselves resistant to bacterial attachment and biofilm development, as was recently exploited by He et al. [127]. Inkjet-based 3D printing was used to fabricate bespoke structures composed of various photoreactive monomers. They found that poly-TCDMDA (tricyclo [5.2.1.02,6]decanedimethanol diacrylate) reduced *P. aeruginosa* biofilm formation by ~99% compared to medical grade silicone. An in vivo study using bioluminescent *P. aeruginosa* showed that such printed implants could reduce the bacteria to negligible levels after 4 days.

Targeted drug delivery using nanotechnology is an alternative approach to specifically targeting the biofilm when releasing drugs. This is often necessary, as conventional antibiotic therapy is difficult, owing to the high doses needed to eradicate biofilms. Aptamers are single-stranded oligonucleotides that fold into a three-dimensional structure and bind their target with high affinity by structural recognition. As such, aptamers against *S. aureus* surface proteins, such as staphylococcal protein A are a promising means to uniquely target proteins expressed exquisitely on the surface of *S. aureus* biofilms. Recently, rifampicin and vancomycin were loaded into liposomes and conjugated to *S. aureus* surface targeting aptamers towards *S. aureus* biofilm eradication [128]. These successfully accumulated around *S. aureus* cells in biofilms, while the liposomes penetrated the biofilm and released sufficient levels of antimicrobial cargoes to eradicate *S. aureus* biofilms in vitro.

Exploiting biofilm characteristics such as local pH, hypoxia, and biofilm composition including the presence of enzymes such as amidase has led to targeted drug delivery techniques to release compounds within biofilms [129,130]. Recently, vancomycin was delivered using DNA NPs modified with pH-responsive lipids [131]. Under physiological conditions, the carrier has a neutral surface charge, while under acidic biofilm conditions, the ionizable lipid gains protons and becomes positively charged, promoting biofilm binding and penetration. These NPs successfully improved bacteria binding and biofilm penetration, and the DNA NPs functioned as an anti-toxin against α-hemolysin. Controlled release of vancomycin prevented biofilm attachment and reduced early-stage biofilm formation within 24 h in vitro. In another study, amphiphilic polymeric vesicles comprising poly(ethylene glycol)-*block*-poly 2-(dimethylamino) ethyl methacrylate (mPEG-*b*-pDEAEMA), loaded with the aminoglycoside antibiotic, apramycin, were developed [132]. These vesicles were also pH-responsive in the low pH of *P. aeruginosa* biofilms and were more efficient at eradicating biofilms compared to free drug in vitro.

Biofilm disaggregation strategies have also focused on weakening the biofilm by targeting the extracellular polymeric substance (EPS), which features key matrix components such as poly-N-acetylglucosamine, extracellular DNA and biofilm proteins using the respective enzymes dispersin B, DNase I and proteinase K. However, this approach required co-administration of antimicrobials to treat dispersed planktonic bacteria. The efficacy of this dispersal approach was demonstrated in vitro using community-associated MRSA LAC biofilms on titanium substrates. The biofilms were sensitive to dispersal by proteinase K and DNase I, indicating that the matrix was composed of proteinaceous material and extracellular DNA. Furthermore, the dispersed cells were susceptible to rifampicin and levofloxacin treatment. [133]. A cocktail of two EPS-degrading enzymes, DNase I and dispersin B, inhibited staphylococcal skin colonization in an in vivo pig skin colonization model [134]. A recent study found that the EPS of Salmonella biofilms is a cooperative trait, with benefits shared among cells, and that EPS inhibition reduces cell attachment and antimicrobial tolerance. As such, the work suggests that targeting cooperative traits such as EPS could be a solution to the problem of antimicrobial resistance [135]. Even though many biofilm disruption technologies are at an early stage of development and lack scientific and clinical evidence to support their safety and efficacy, it is salient to note that implant removal represents the most effective means of eradicating clinical infections with a biofilm aetiology, underscoring the need for innovative technologies.

## 5. Antimicrobial Surface Coatings

Antimicrobial agents may be incorporated on the device either directly (physically or chemically by grafting) or in a coating platform to achieve temporal control over local presentation at the target site [136,137,138]. Antimicrobial effects owing to nanotopography, and surface engineering are described in Section 6 and Section 7 (Figure 2).

### 5.1. Direct Antimicrobial Application to the Implant Surface

Local antimicrobial presentation at the biological interface offers a number of advantages compared to the standard approach of systemic delivery including (i) the potential to use lower drug doses due to localized targeting, (ii) greater control over toxicity and bioavailability, (iii) reduced likelihood of promoting antibiotic resistance in one respect because delivery is proximal to infection site, (iv) the possibility to achieve extended drug release and to combine drugs with different kinetics, (v) controlled release from surfaces of combination devices directly to the target site, (vi) avoidance of systemic drug exposure, and (vii) direct mitigation of device-related infection [138]. However, the merits of strategies based on the release of antimicrobials, particularly drug cargoes, have been debated due to poor control over drug kinetics, with many systems displaying burst and short-term release profiles, whilst prolonged subtherapeutic drug levels carry the risk of promoting drug resistance.

One of the earliest approaches to preventing ODI involved coating intramedullary nails with a combination of antiseptics (chlorhexidine and chloroxylenol) [139]. The nails, which were coated with antiseptics using a dipping technique, offered substantial protection against *S. aureus* in a rabbit model of open tibial fracture, without releasing the antiseptics into the serum [139]. Meanwhile, rifampicin and minocycline-coated titanium (Ti) implants were implanted into the femoral medullary canal of rabbits and reduced bacterial colonisation and osteomyelitis for one week, compared to uncoated implants [140]. Teicoplanin was directly sprayed onto Ti wires [141]. After 24 and 48 h, inhibition of *S. aureus* growth was observed in vitro. Furthermore, in an in vivo rabbit model, there was no growth of *S. aureus* on the Ti wires, on the bone tissue or in the blood cultures [141].

One common drawback to the abovementioned examples is the lack of any “controlled release” aspect to the systems. An alternative approach involved covalent conjugation of an antibiotic to implants [142], whereby antibiotic is not eluted into the systemic circulation but prevents attachment of bacteria to the implant. When vancomycin was covalently tethered to the surface of Ti rods, attachment of *S. epidermidis* bacteria was reduced and biofilm formation was inhibited compared to control Ti rods [143]. This system exerted specific activity against Gram-negative bacteria and maintained its activity after exposure to serum proteins and after repeated exposure to high bacterial loads [143]. Such an approach minimizes the risk of systemic toxicity and provides a longer duration of protection. Antibiotics have also been covalently attached to implant surfaces via a linker such as ethylene glycol [144]. Vancomycin was covalently tethered to Ti beads via an aminoethoxyethoxy acetate linker and inhibited biofilm formation and proliferation of *S. aureus* [144]. Antimicrobials have also been directly conjugated to haemodialysis catheters. For example, polyurethane catheters were coated with selenocyanatodiacetic acid (an organoselenium antimicrobial) by covalent attachment, resulting in reduced biofilm formation both in vitro and in vivo in a mouse model of chronic biofilm infection for up to five days [145].

### 5.2. Antimicrobial Delivery from Polymer Coatings

Non-degradable polymers, most notably PMMA beads, are extensively used in the clinic to prevent implant-related infections. Several antibiotic-loaded PMMA bone cement products have been approved by the FDA, including Simplex with tobramycin and gentamicin (Stryker Howmedica Osteonics) [146] and SmartSet GHV with gentamicin(DePuy Orthopaedics) [147,148]. However, there are some critical disadvantages associated with such non-degradable polymers including the requirement of a follow-up procedure for removal of the beads and the potential for resistance due to sustained release of subtherapeutic levels of antibiotics [149]. Biodegradable polymers containing various antibiotics have been investigated for coating implants, with some demonstrating considerable success in experimental models. A key advantage of biodegradable polymer coatings is that they can be designed to accurately release the antibiotic over a specified period, often exhibiting two phases of drug release. The early “burst” release phase is useful for killing the microorganisms found in the implant area before they settle and create a biofilm [150,151]. The second phase of drug release is slow and sustained, ranging from several days to weeks, and this prevents the development of infections at the implant site during healing. The burst effect is obtained due to diffusion of drug molecules located on the surface and in polymer layers close to the surface, while the continuing release is obtained due to drug diffusion and polymer degradation [151].

The most widely investigated biodegradable polymers are the poly(α-hydroxy esters) including P_L_LA, poly-D-L-lactic acid (P_DL_LA) and polylactic-co-glycolic acid (PLGA). PLA and PLGA have been used to coat implants with a whole range of antibiotics as well as antiseptics and the efficacy of these in infection prophylaxis has been assessed both in vitro and in vivo in several animal models including rat and rabbit models of induced implant-related infection [152,153,154]. These polymers have numerous benefits, principal among them includes their biocompatibility, and the potential for tuneable drug release depending on monomer type and ratio (in copolymers). The polymers, which are used in a number of currently marketed parenteral depots and implants, offer the ability to control the release of a range of cargoes types including hydrophilic and hydrophobic drugs, both small molecule cargoes and larger biological molecules [155], [71]. Selected examples of antibiotic containing P_L_LA, P_DL_LA and PLGA coatings are detailed in Table 2. In one instance, the in vitro and in vivo results were so promising that eight patients with open, tibial fractures were treated with antibiotic polymer-coated intramedullary nails, and after one year there were no signs of soft-tissue reactions or systemic infection [156].

Although these polymers have been extensively investigated for the purpose of delivering antibiotic therapy for the local treatment of infections in bone, there has been some debate surrounding their use. Several studies have questioned the biocompatibility of PLA and PLGA due to the release of acidic by-products of degradation, which have been attributed to a delayed adverse biological response often reported in humans [157,158]. The drop in pH associated with the acidic by-products may also adversely affect the functionality of some antimicrobial compounds whose efficacy is dependent on maintenance of pH in the physiological range [159]. Despite these concerns, this technology has been successfully translated to the clinic by DePuy Synthes to impede bacterial colonization on the implant surface. The marketed tibial nail device “Expert PROtect” is designed to deliver gentamicin sulphate from a thin, resorbable P_DL_LA coating (approx. 50 µm) immediately after implantation for a duration of approximately 2 weeks. The device is deemed suitable in cases where an increased risk of infection exists, and bacteria are gentamicin sensitive. Such scenarios include all open fractures (Gustilo–Anderson Grade I to III) and secondary nailing (exchange of nail to nail or external fixator to nail) in cases where revision surgeries are necessary due to infections and instances of polytrauma [160].

Alternative polymer coatings that have been investigated include poly(trimethylene carbonate) (PTMC) [161], polycaprolactone (PCL) [162] and polyurethane (PU) [163]. These may offer some advantages over the traditionally used P_L_LA and PLGA carriers. For example, degradation of PTMC does not yield acidic by-products, and its potential suitability as a carrier for antibiotics including vancomycin and gentamicin has been shown [161]. Polyelectrolyte films, composed of biodegradable polymers, assembled in a layer-by-layer process and incorporating drugs such as antibiotics, have also been investigated as implant coatings [164]. In a proof-of-principle study, a biodegradable coating composed of polyelectrolyte (cationic β-amino ester and anionic polyacrylic acid) multilayers with gentamicin was applied to Ti dowels and investigated for antibacterial efficacy in a rabbit model of implant-related osteomyelitis [164]. In vitro, the films exhibited a burst release (two-thirds of antibiotic released within three days), followed by a sustained release for up to 5.5 weeks, with the concentration of gentamicin exceeding the minimum inhibitory concentration (MIC) for *S. aureus* throughout the study. Furthermore, gentamicin-loaded films significantly inhibited growth of bacteria compared to uncoated implants [164]. Polyelectrolyte films have also been investigated for controlled release of AMPs, such as ponericin G1, which is active against *S. aureus* [165], and a combination of anti-inflammatory and antibacterial drugs [166]. The latter approach may be of particular interest when considering the co-existence of inflammation and infection in osteomyelitis. Diclofenac and gentamicin were successfully loaded into polyelectrolyte films and spray-coated onto a range of medical devices including bandages and intraocular lenses, with therapeutic efficacy shown against *S. aureus* in vitro [166].

As an alternative to the manufacture of antimicrobial coated implants, the delivery of high concentrations of antibiotics to the surgical site during the perioperative period has also been proposed using antibiotic-loaded thermo-responsive gels. Other advantages of this approach include flexibility in terms of spatial distribution and applications in complex wounds, e.g., open fracture care. A biodegradable, gentamicin-loaded, thermo-responsive poly(N-isopropylacrylamide)-grafted hyaluronic acid (HApN) hydrogel prevented implant-related infection in a rabbit osteosynthesis model [167] and did not adversely affect fracture healing [168]. The early and local application of a gentamicin-loaded hydrogel was demonstrated to be superior to perioperative systemic prophylaxis [169].

There has been debate about the long-term efficacy and risk of resistance associated with coated implants. Meta-analysis of animal studies evaluating the performance of coated titanium implants to prevent *S. aureus* infections concluded that active coating of titanium implants produced positive data against both MSSA and MRSA [95], while patient data has shown that antibiotic-coated implants are beneficial in the management of open tibial fractures in patients at high risk of infection, reducing infection by 75% [170,171].

**Table 2:** Antimicrobial delivery systems based on PLA and PLGA coatings for metal implants. The table highlights polymer type, antibiotic/antiseptic, implant substrate, in vitro or in vivo model, release kinetics and experimental outcome.

Polymer	Antimicrobial	Implant	Model	Release Kinetics	Outcome	Reference
P_DL_LA	Norvancomycin	Stainless steel plate	Rabbit tibia fractures, inoculated with *S. aureus*	Sustained release above minimum inhibitory concentration for up to 28 days	Significant reduction in infection rate (32% compared to 92% for uncoated plates)	[152]
PLGA	Vancomycin Cefuroxime	Ti alloy discs	*S. aureus* culture in vitro	Effective antibiotic release duration from 5 to 17 days depending on antibiotic and concentration	Up to 17 days antibiotic release from optimal double layer formulation	[154]
P_L_LA	Rifampicin and fusidic acid; octenidin and triclosan (antiseptics)	Ti plates	Rabbit tibia model, inoculated with *S. aureus*	Release of 60–62% within 1 h, then sustained release for at least 42 days	Significant reduction in infection rate (17% for antibiotic and antiseptic groups compared to 83% for control groups)	[153]
P_DL_LA	Gentamicin and/or teicoplanin	Stainless steel and Ti alloy K-wires	*S. epidermidis* culture in vitro	Initial burst release within 6 h, then sustained release for at least 96 h	Reduction in adhesion of viable bacteria to undetectable levels with either or both antibiotics	[172]
PLGA	Gentamicin	Stainless steel fracture plates	*S. aureus* culture in vitro	Initial burst release, with sustained high levels for 3 weeks (for the 20% gentamicin coating)	Significant reduction in bacterial growth compared to uncoated implants)	[173]
P_DL_LA	Gentamicin	Ti K-wires	*S. aureus* induced intra-medullary infection	60% release within 24 h, then sustained release over 6 weeks (from previous study)	Significant reduction in histological and radiological signs of infection in treated groups compared to control groups	[156]
P_DL_LA	Gentamicin	Ti K-wires	*S. aureus* induced intra-medullary infection in rats	80% gentamicin release within 48 h (demonstrated in previous study)	Significant reduction in radiological signs of infection compared to control group	[174]
P_DL_LA	Gentamicin	Ti K-wires	*S. aureus* induced intra-medullary infection in rats	60% release within 24 h, up to 90% released in the following 6 weeks (demonstrated in previous study)	Significantly lower histological infection score (with or without systemic gentamicin) compared to other groups	[175]
PLA	Chlorhexidine	Ti plates	*S. aureus* culture in vitro	Rapid release during first day, followed by slower release up to 14 days	Greatest antibacterial effects with lowest % PLA coating	[176]
Copolymer of glycolide, caprolactone, trimethyl carbonate, lactide	gentamicin, triclosan or combination	Stainless steel plates, covered with polymer sleeve	Adult sheep	Gentamicin: 50% release within 24 h, then sustained release over 2–3 weeks Triclosan: slow release over 2–3 weeks	Tissue biocompatibility and normal bone healing demonstrated	[177]
P_DL_LA	Gentamicin	Ti K-wires	Rat with intramedullary implant	Burst release within 1 h, then gradually reducing levels over 7 days	Significantly reduced bacterial adhesion compared to uncoated wires	[178]
PLGA	Gentamicin	Ti coupons	Staphylococcal cultures in vitro	Release of 90% within 24 h, then short, sustained release over 4 days	Greater antibacterial activity compared to uncoated coupons	[179]

K (Kirschner), P_L_LA (poly-L-lactic acid), (P_DL_LA (poly-D-L-lactic acid), PLGA (polylactic-co-glycolic acid)), *S. aureus (Staphylococcus aureus*), *S. epidermidis (Staphylococcus epidermidis*), Ti (titanium).

### 5.3. Antimicrobial Delivery from Inorganic and Ceramic Coatings

Ceramic coatings for implants, including tri-calcium phosphate and HA, have also been investigated for antimicrobial delivery. These bioactive materials exhibit compositional properties similar to native bone, are inherently osteoconductive and offer the added benefit of improved integration at the implantation site. This is particularly desirable in the case of devices intended for permanent implantation where integration with host tissue is key to long-term device success. Calcium phosphate coatings have been used as a platform to present an array of antibacterial agents including drug molecules gentamicin [180] and vancomycin [181], metal ions, e.g., Ag [182] and biological cargoes e.g., AMPs [116]. Examples of antimicrobial agent incorporation into inorganic coatings are provided in Table 3.

Extensive drug incorporation into HA coatings and slower release profiles have been observed for more acidic antibiotics, including cephalothin and carbenicillin, likely due to chelation of calcium ions in the HA by the carboxylic groups in these antibiotics [5]. Meanwhile, basic antibiotics, such as gentamicin, were incorporated to a lesser extent and were rapidly released from the coating. Another factor found to influence antibiotic delivery from HA was the porosity of the carrier material, with higher levels of antibiotic adsorbed onto microporous HA, as well as prolonged release [5]. Antibacterial effects from HA coatings have been shown in vitro to occur rapidly within 1 [181] to 24 h [183] and have in some instances been reported to last for 28 to 70 days, depending on the technique of application and the amount of HA in the coating [184].

There are several reports of antibiotic-containing ceramic coatings for implants in the literature, showing favourable outcomes in vivo. One of the earliest studies investigated gentamicin-loaded HA coating of stainless-steel K-wires [180]. Infection rates were significantly reduced with these antibiotic ceramic-coated implants compared to standard HA-coated implants in a rabbit intramedullary *S. aureus* infection model. In a similar animal model, tobramycin-loaded peri-apatite-coated Ti implants showed significantly reduced levels of infection compared to controls [185]. Introduction of the gentamicin antibiotic to a calcium phosphate-based implant coating at the intraoperative stage using a dipping process immediately prior to implantation was also investigated as a simplified strategy for targeted delivery of antibiotics. In vivo evaluation using a rat proximal tibia model showed that device-related infection was prevented in seven out of eight animals at 7 days in contrast to infection in all drug-free controls [186].

Sol–gel coatings have also been investigated as an alternative mechanism for time-controlled local release of antibiotics from orthopaedic implants [187]. For example, a silica sol–gel containing vancomycin was coated onto Ti alloy rods and antimicrobial activity was assessed in a rat osteomyelitis model [188]. The authors reported that bacterial colony counts were reduced and clinical signs of osteomyelitis were minimal compared to non-coated implants, although it appeared that the antibiotic was fully eluted by 2–3 weeks [188]. In another study, nitric oxide (NO) was incorporated into *N*-(6-aminohexyl)-aminopropyltrimethoxysilane/butyltrimethoxysilane (AHAP3/BTMOS) sol–gel and coated onto stainless steel slides [189]. The rate of NO release was found to be dependent on temperature and adhesion of *S. aureus* was significantly lower for NO releasing sol–gels compared to uncoated slides in vitro [189].

**Figure 4:**
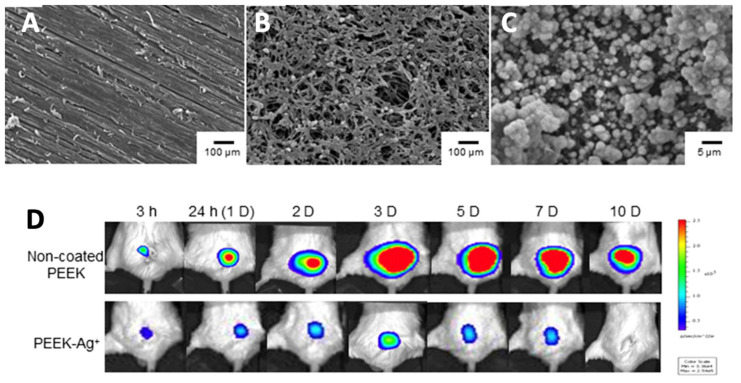
Example of an ionic silver antimicrobial coating immobilized onto hydroxyapatite covering a polyether–ether–ketone (PEEK) implant coating. The surface of PEEK (**A**) was immersed in 98% concentrated sulfuric acid (H_2_SO_4_) for 10 min (**B**), after which a porous configuration was observed on the surface by SEM. (**C**) Hydroxyapatite aggregates were homogeneously coated on the PEEK coated with immobilized Ag^+^ ions (PEEK-Ag^+^). (**D**). Sequential analysis of the bacterial bioluminescence in the mouse soft tissue infection model, where a non-coated PEEK of PEEK-Ag^+^ plate was placed into the superficial gluteus muscle of mouse, followed by inoculation with a bioluminescent strain of *S. aureus*. The bacterial photon density was measured at 3, 12 and 24 h, and then each day until 10 days after the operation). Modified and reproduced from [190] under CC BY 4.0 http://creativecommons.org/licenses/by/4.0/ (accessed on 27 July 2022).

Several methods have been investigated to deposit antimicrobial ion coatings on implant substrates. Bose’s group utilized plasma-spraying to deposit an HA coating with a ternary dopant system composed of ZnO to induce osteogenesis, SiO_2_ to induce angiogenesis, and Ag_2_O to provide secondary infection control [182]. A silver-immobilized HA PEEK coating on Ti implants was developed by immobilizing ionic silver onto a HA film via inositol hexaphosphate chelation, using a series of immersion and drying steps performed at low heat. The coating enabled complete eradication of a bioluminescent *S. aureus* infection and biofilm formation in a soft tissue model in murine superficial gluteus muscle (Figure 4) [190]. A novel, ambient temperature grit-blasting process, CoBlast™ has been used to deposit HA- and metal ion-doped apatite coatings on Ti substrates. [70,107]. Both single-ion Ag, Zn and Sr and binary dopants (Ag-Sr) have been investigated. Silver-substituted HA coatings on Ti demonstrated good antibacterial activity (eluted and immobilised ion) against several clinical isolates including MRSA and methicillin-sensitive *S. aureus* although it poorly inhibited *S. epidermidis.* The co-substituted Ag-Sr apatite surface did not show enhanced osteoblast or anti-colonising properties compared with the single dopant Sr and Ag apatite surfaces [191].

**Table 3:** Antimicrobial-containing inorganic coatings on orthopaedic implants. Table highlights the carrier type, antimicrobial, implant type, in vitro or in vivo model, antimicrobial release kinetics and experiment outcome.

Carrier	Antimicrobial	Implant	Loading and Release Kinetics	Model	Outcome	Ref.
Silica sol–gel films	Triclosan	Percutaneous external fixator pins—stainless steel 316L rods	Continuous release in vitro with 33% of original load by 8 weeks.	Percutaneous tibial implant rabbit model, inoculated with *S. aureus* (ATCC™ 25923)	No infection in animals with pin implants coated with sol–gel 20% triclosan in contrast to uncoated implants. Normal bone tissue ingrowth observed at 4 weeks in coated implant model.	[192]
Silica sol–gel films	Vancomycin + farnesol (adjuvant)	Ti alloy rods and K-wires	Drug release from 5-layer thin films is concomitant with film degradation over 6 days.	In vitro bacterial challenge (i) MSSA (ATCC 25923) 5 mL 1 × 10^6^ CFU/mL (rods), (ii) MRSA (ATCC 33591) 5 mL 1 × 10^4^ CFU/mL (wires)	Bactericidal effect impacted by drug loading and farnesol on K-wires. Bacterial (MRSA) counts 1.11 × 10^3^ and 1.56 × 10^2^ CFU for Vancomycin 10% *w*/*w* and 20% *w*/*w* loading, respectively. Further reduction to 2.44 × 10^2^ CFU when farnesol added to 10% vancomycin.	[193]
TNT	Vancomycin	Ti rods	Drug loading by lyophilization, with rapid release −58% release within 15 min.	Femur Sprague–Dawley rat model—intramedullary implantation of rod contaminated with 0.1 mL of 1 × 10^8^ *S. aureus* (29213 ATCC)	Agar plate and clinical assessment at 30 days showed all animals receiving vancomycin-containing nanotubes were infection-free, while 11 out of 12 drug free-TNT controls were infected.	[194]
Hybrid coating—TiO_2_ and PDMS	Ag	PEEK discs	Release rate depends on Ag doping and ratio of coating constituents.	*S. aureus* (ATCC 25923) and *S. epidermidis* (ATCC 35894) 5 × 10^6^ CFU/mL used for Kirby–Bauer testing and biofilm growth studies.	Kirby–Bauer testing showed greater zones of inhibition for higher Ag loading, with similar results for both bacterium types. SEM analysis revealed small colonies of *S. aureus* for the lower Ag loading compared to controls. Colonies were absent in higher Ag loadings.	[195]
HA	Ag	PEEK	Ionic Ag is immobilized via inositol hexaphosphate chelation	A non-coated PEEK or PEEK-Ag+ plate was placed into the superficial gluteus muscle of mouse, followed by inoculation of bioluminescent S. aureus (1 × 10^11^ CFU/mL)	Mean bacterial photon intensity decreased after 8 days and reduced to background level at day 10 in the PEEK-Ag^+^ model, compared to non-coated PEEK where strong photon intensity was still observed at day 10.	[190]
HA	Zn, Ag and Sr	Ti (Grade 5, Ti-6Al-4V)	At 30 days <10% of Sr and Zn but > 90% of the Ag released	Antimicrobial activity of the released ions and anti-colonizing potential of the surface using modified ASTM E2149-01 and *S. aureus* ATCC 1448	MG-63 osteoblast cells cultured on the Sr apatite surfaces displayed the highest metabolic activity using the MTT assay. Ion release and direct surface contact important for antibacterial effects. Ag-substituted apatite produced superior biofilm inhibition compared to Sr and Zn substituted apatite surfaces.	[107]
Ti	Zn	Ti	For 1 cm^2^ coatings, total Zn loading ranged from 1.2 to 60.2 μg, depending on time of hydrothermal treatment in Zn solution and voltage used during NT fabrication	Incubation of implants in *S. aureus* and viable bacteria in suspensions collected from samples were evaluated by spread plate method.	Inhibition of adherent and planktonic bacteria was greatest for the coatings with the highest Zn content. Inhibition was greatest at day 1 and decreased at days 4 and 7.	[196]
Three-layer. Outer and inner vanco-mycin loaded in vaterite. Middle layer IL-12 containing liposomes embedded in alginate.	Vancomycin	Ti	Vaterite coating released 100% of vancomycin within ~2 days.	In vitro: soaking of different layers of the coating with 1 mL of sterile LB broth and inoculated with 200 μL of LB broth with a concentration of 1 × 10^6^ CFU/mL ATCC 25923 (MSSA) and ATCC 43300 (MSSA) bacterial strains. In vivo: rats were inoculated with MSRA in the tibial platform and Ti alloy screw was implanted in the tibial channel, re-injection of MSRA after two weeks and injection of 0.1 mL of LB broth containing 1 × 10^6^ CFU/mL bacteria in the bone marrow cavity.	In vitro: complete three-layer sandwich yielded a bacterial death ratio of ~100% of ATCC 25923 by day 7; complete three-layer sandwich yielded a bacterial death ratio of ~100% of ATCC 43300 by day 7. In vivo: bacterial colonization in the bone tissue reduced in the three-layer sandwich compared to control.	[197]
Biphasic calcium phosphate	Vancomycin Tobramycin	Drug-loaded biphasic calcium phosphate granule complex with additive antibiotic powder in gypsum binder, coated with PLGA	Human trial composing of 43 patients with previously diagnosed chronic osteomyelitis subjected to prosthesis removal (if present), debridement of necrotic tissue, and 20–40 g of cements containing beads were implanted into bone defects before primary wound closure.	Serum concentrations of vancomycin and tobramycin decreased gradually from ~ 20 μg/mL to 0 μg/mL over 14 days.	Higher success rate, faster sepsis control and bone regeneration achieved compared to PMMA cement and parenteral antibiotic therapy.	[198]

Ag (Silver), AMP (antimicrobial peptide), ATCC (American Type Culture Collection), HA (Hydroxyapatite), IL-12 (Interleukin-12), K (Kirschner), NTs (nanotubes), PEEK (polyether–ether–ketone), PDMA (polypolydimethylsiloxane), PDMA (Poly (N,N-dimethyacrylamide)), PAPMA (Poly N-(3-aminopropyl) methacrylamide)), SEM (scanning electron microscopy), Sr (strontium), *S. aureus (Staphylococcus aureus*), *S. epidermidis (Staphylococcus epidermidis*), Ti (titanium), TiO_2_ (titanium dioxide), TiO_2_ nanotubes (TNT), Zn (zinc).

## 6. Nanotechnology and Infection Control

There is increasing interest in the field of nanotechnology to address both existing challenges in the field of medicine and to advance the scientific field beyond what is currently possible. The diversity of materials and the ability to tailor their functionality and properties has extended the repertoire of applications to include drug delivery, diagnostics and immunization across a range of clinical applications including cancer therapy, diabetes, bone-healing, and cardiovascular applications [199]. Nanoparticles (NPs) have at least one dimension less than 100 nm, often exhibiting distinctive physicochemical properties and high surface–volume ratios at the size domain that contribute to their efficacy. In the context of infection control, antimicrobial nanomaterials, nanosized drug carriers and nanostructured surfaces are of particular focus because they offer the prospect to increase targeting and eliciting multiple mechanisms simultaneously to combat microbes, thereby decreasing the likelihood of resistance developing [200,201]. However, the toxicity profile associated with long-term exposure to many nanosized materials and drug carriers needs to be demonstrated, particularly if intended for repeated use [202]. Bactericidal nanostructuring of materials has emerged as a trend in nanoscale approaches to infection control on the surface of implants and is discussed in Section 7.

### 6.1. Antimicrobial Nanomaterials

The intrinsic antimicrobial activity of a range of nanostructured materials including polymers (chitosan), metals (Ag, gold, copper) and metal oxides (Al_2_O_3_, ZnO, TiO_2_) together with the capacity to modify the functionality of the implant surface (surface charge, topography) or enhance the effectiveness of antimicrobial cargoes highlights the versatility and promise of their usage to prevent or treat device-related infections. The mechanism of antibacterial action is not completely understood in all cases but is generally understood to stem from the disruption of the bacterial cell membrane, induction of oxidative stress, interference with cell processes including energy transduction, enzyme activity and DNA synthesis. The composition and physicochemical properties of the nanostructured material in addition to bacteria type impact this activity [96,203]. Of the materials listed, some are at an earlier stage of investigation for the purpose of infection prevention, whilst the use of Ag metal and Ag ions for antibacterial purposes is particularly well-established. The mechanism of action is thought to differ depending on whether Ag is present in the ionic or NP form, or if it is surface immobilized [103]. Concomitant release of ionic silver from Ag NPs can augment the bactericidal activity [204]. Although this resurgence in interest stems primarily from broad spectrum activity of Ag and the need for strategies to treat multi-resistant pathogens, it has been facilitated by the advance of nanotechnology approaches allowing application of pure Ag in NP form, thereby enabling efficacy at smaller doses and decreased toxicity [205]. One group recently functionalized porous Ti implant with silver and zinc NPs by plasma electrolytic oxidation. The surfaces maintained release of silver and zinc ions for at least 28 days and implant surfaces containing 75% silver and 25% zinc NPs fully eradicated MRSA bacteria in vitro and in an *ex vivo* murine femora models. The antibacterial synergism of silver and zinc enabled a 120-fold reduction in the amount of silver ions required for such effects [206]. Recently, a hybrid nanocoating with different modes of action was designed and tested to prevent implant infections. The hybrid nanocoating deposited on etched Ti surfaces featured self-assembled AMP (dGL13K) nanofibrils decorated with AgNPs. The hybrid nanocoating had higher in vitro antimicrobial potency against MRSA compared to *P. aeruginosa,* and the hybrid coating was superior to both AgNP and AMP coatings [207].

Other NPs composed of TiO_2_ and ZnO also mediate their activity by damaging the cell membrane. TiO_2_ has been used to impart antibacterial properties in addition to controlling drug release from biomedical device surfaces and has been reviewed elsewhere [208,209]. Its activity is dependent on photocatalytic activation by irradiation with near-UV and UVA to generate reactive oxygen species (ROS), but efficacy apart from photoinduced activity has also been reported [96]. Antibacterial efficacy arising from surfaces based on photocatalytically activated anodized TiO_2_ [210] has been demonstrated. Titania nanotubes are also of interest because control over their geometrical and physiochemical properties is reputed to endow the TiO_2_ structures with inherent antimicrobial potential [86]. Additionally, the surface chemistries of such TiO_2_ nanotubes can be modified in a variety of ways, including covalent attachment of PEG [211].

### 6.2. Nanotechnology Approaches for the Delivery of Antimicrobials

Nanoparticulate drug delivery platforms offer the advantage of improving drug targeting and pharmacokinetics, and the opportunity to sustain drug release whilst reducing adverse drug events. Owing to their size they possess a number of properties, which has prompted interest in their infection control potential including the possibility to facilitate high levels of drug loading [150], of a range of antimicrobial cargoes and their high surface-to-volume ratio makes them amenable to surface functionalization and an increased potential to interact with pathogens [212]. Materials that can be used for NPs can be classified based on their character and origin, e.g., organic/inorganic, or natural/synthetic. A variety of NP formulations have been investigated to enhance drug delivery including those composed of polymers, dendrimers, metals and lipid constituents (solid lipid NPs, liposomes) [155]. Nanoparticles composed of polymeric materials are of particular interest as they can be tailored to release the drug cargo either continuously due to the biodegradable nature of the carrier or in response to specific stimuli. For example, NPs synthesized from poly(ethylene oxide) macromonomers, linked to gentamicin by means of a pH sensitive bond, were tethered to Ti disks, yielding a “smart” system, in order to release the antibiotic in the low pH conditions of an infection [150]. Inhibition of bacterial growth was maximal at the lowest pH (pH = 4) after 48 h [150]. In another study, PEG/PLGA co-polymer NPs delivered in a temperature-responsive hydrogel were used to encapsulate teicoplanin and were implanted into the femurs of an osteomyelitis rabbit model, showing equivalent therapeutic efficacy to traditionally used PMMA beads, with the advantage of biodegradability [213]. Mesoporous silica nanoparticles (MSNs) were investigated for multiple antibiotic delivery in a bid to achieve a synergistic therapeutic effect [214]. The loading capacity for moxifloxacin was 54.18 ± 1.86 μg/mg MSNs and 37.88 ± 3 μg/mg MSNs in the case of rifampicin. Drug release was biphasic with an initial, rapid release phase followed by sustained release. Coating of particles with gelatine and colistin decreased moxifloxacin release from 100% to ~60% at 24 h, while rifampicin release decreased from 100% to 40% at 24 h. In vivo studies were conducted in New Zealand white male rabbits implanted with cylindrical Ti-6Al-4V implants. The implants were infected with MRSA strain SAP231 for 24 h to achieve a bacterial density of 6.82 CFU/cm^2^ prior to placement. After 2 days, the animals received multi-drug-loaded (moxifloxacin MSN and rifampicin MSN) coated MSNs or controls. After 24 h of treatment, the SAP231 quantity per gram of femur was reduced to ~3.5 log_10_ CFU/g of femur in the treated group compared to ~4.5 log_10_ CFU/g of femur in the control.

**Figure 5:**
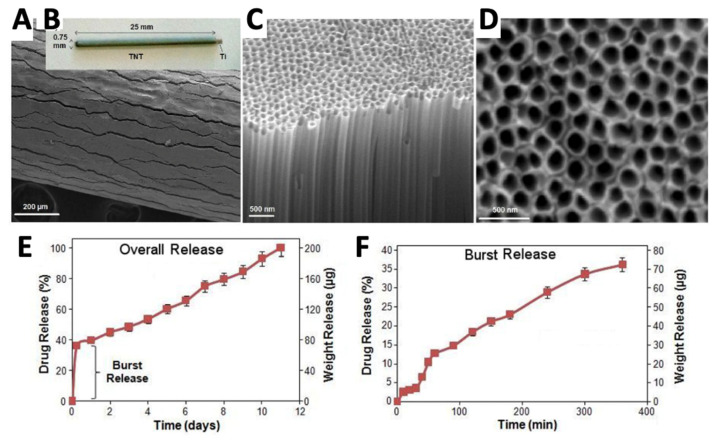
SEM images of titania nanotubes (TNT) grown on Ti wire using the anodization technique. (**A**) The top surface showing cracks, (**B**) the entire structure showing TNT on Ti wire with dimensions, (**C**) the cross-section showing array of TNTs and (**D**) the hollow nanotubes. (**E**) Overall release and (**F**) burst release of gentamicin (corresponding to the first 6 h of fast diffusion of drug) from TNT-Ti wire. Modified and reproduced from [216] CC BY 2.0 http://creativecommons.org/licenses/by/2.0 (accessed on 18 October 2022).

Titania nanotube arrays on the surface of Ti implants have also been investigated as reservoirs for antibiotics with the aim of minimizing bacterial adhesion and infection [215]. The release profile of gentamicin from Ti nanotubes on Ti Kirschner wires was, in one study, found to be similar to that from polymeric coatings, with an initial and rapid burst release of antibiotic, followed by sustained release over the next 11 days [216] (Figure 5). Furthermore, the topography of the surface could be modified by altering the method of preparation, with the greatest release of the antibiotic, in this case cefuroxime, by the nanotubular surfaces compared to the nano-smooth surfaces, likely due to the higher loading capacity [217].

## 7. Controlling Bacterial Infection by Preventing/Minimizing Bacterial Adhesion

**Figure 6:**
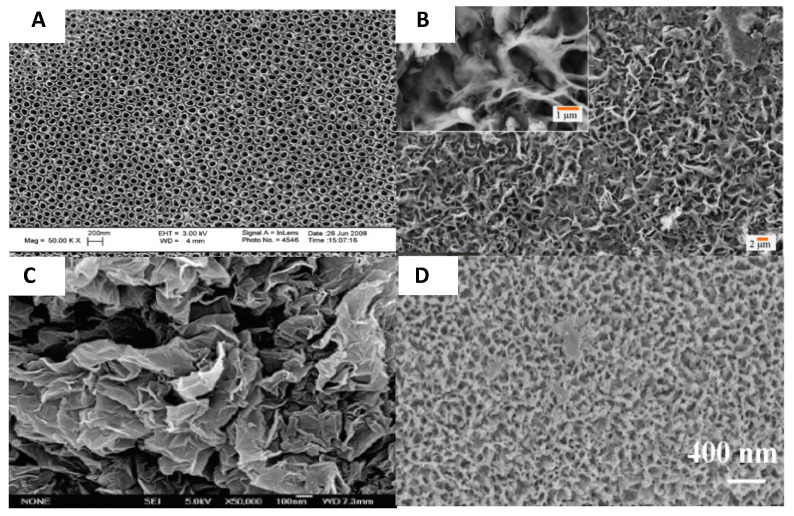
Architectural representation of various bactericidal or repellent surface engineering strategies outlined in Section 7. (**A**) Nanotubular Ti after anodization, adapted with permission from [220]. (**B**) Multilevel (micro- and nano-) roughened aluminium alloys adapted from [221] under CC BY 4.0 http://creativecommons.org/licenses/by/4.0/ (accessed on accessed on 18 October 2022). (**C**) Reduced graphene oxide (rGO) nanosheets on silicon wafer adapted with permission from [222]. (**D**) Hyperbranched poly-L-lysine coating on Ti implant adapted with permission from [223].

Numerous factors influence bacterial adhesion and colonisation of the implant surface, including properties of the pathogen (genus and species, gram positive or gram negative, shape, and adhesion expression) and environmental conditions (pH, host proteins, volume, viscosity and shear properties of physiological fluids) [218]. It is recognised that the implant surface has a key influence on bacterial adhesion, and manipulation of its features may be employed as a drug-free strategy to reduce ODI. Despite the lack of quality data in the literature elucidating the exact mechanisms of bacterial adhesion, there are numerous studies dedicated to engineering physicochemical properties of the surface (Figure 2) including hydrophobicity/hydrophilicity, surface charge and functional groups, in addition to controlling surface topography at multiple length scales (micro and nano) to control bacterial adhesion and biofilm formation [219]. Examples of drug-free, surface-mediated antimicrobial implants are described in Table 4 and illustrated in Figure 6.

### 7.1. Surface Topography

Bactericidal nanostructure geometry is an emerging area of research in anti-infective orthopaedic implants and offers a promising alternative approach to combating bacterial contamination. Physical alteration of material surface topography obviates the need for antibacterial agents and their associated limitations, including inevitable leaching and depletion of drug and the promotion of antimicrobial resistance. Surface roughness is well known to influence interactions between bacteria and the surface [224], but results have varied, likely due to differences in topography, the size and shape of surface features, surface chemistry and bacterial species [225]. The relationship between nanostructured surfaces and bacterial attachment may vary depending on the exact nanoscale topographical features. One study observed decreased adhesion of *S. aureus, S. epidermidis* and *P. aeruginosa* on nanoroughened surfaces produced by electron beam evaporation in contrast to nanotubular and nanotextured Ti surfaces produced by anodization processes [220].

Bactericidal topography approaches have taken inspiration from nature, including the cicada wing, which can kill bacteria such as *E. coli* and *P. aeruginosa* [226]. The mechanism of bactericidal action was attributed to mechanical rupture of bacterial cell membranes induced by cell interaction with the wing’s nanotopography. The *Psaltoda claripennis* cicada wing is composed of spherically capped nanopillar surface structures that exhibit bactericidal properties against Gram-negative bacteria. The chemically inert nanopillars exert their bactericidal effect via a physical mechanism. It has also been postulated that adsorption of bacterial cell membrane onto the cicada wing leads to a stretching effect, leading to cell membrane rupture and death [227]. It seems that bacteria initially sink down onto the wing nanotopography, spread between nanopillars, then suddenly experience a short downward displacement, indicating lethal rupture of the cell. However, it is not effective against Gram-positive species, likely because this species of bacteria has thick peptidoglycan layers, hence generating a stress-bearing and more rigid surface. The nanopillar architecture of the cicada wing has inspired research teams to recapitulate such nanotopography on implant surfaces to prevent bacterial contamination. Linklater et al. (2021) recently conducted and in-depth review on the different physicochemical and mechanical interactions between nanostructured surfaces and bacteria that are purported to prevent bacterial attachment and elicit bacterial killing [228].

**Figure 7:**
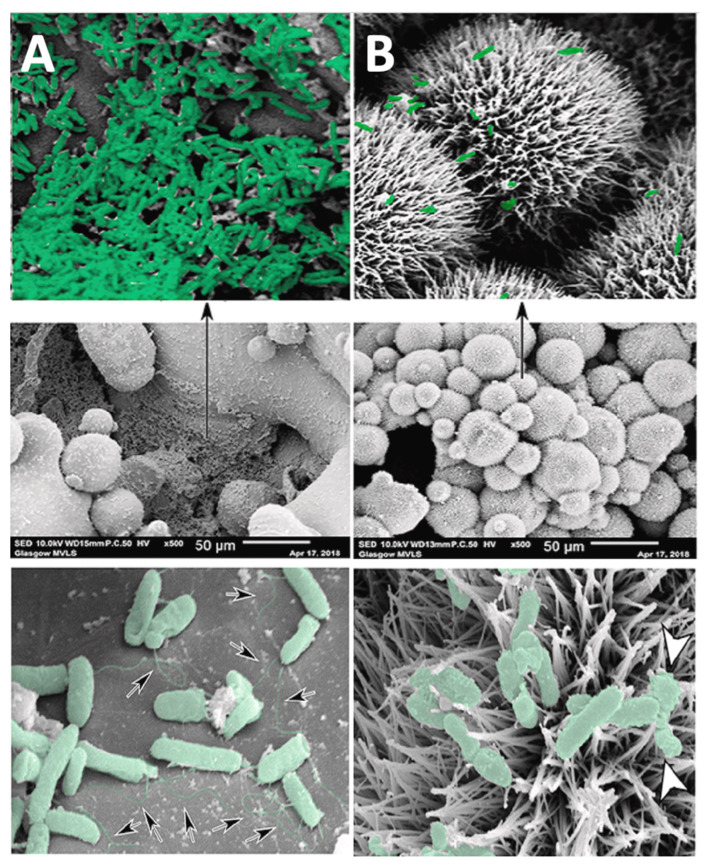
SEM of *P. aeruginosa* response to the (**A**) D900 uncoated flat control (top, middle rows). Flagella were observed on the control samples (arrows) (bottom row in column **A**). (**B**) *P. aeruginosa* response to the 90 s pPEA-2 hr fibronectin (FN), bone morphogenetic protein-2 (BMP2)-coated D900 surface (top, middle rows, column **B**). Flagella were absent in bacterial cells observed on 90 s pPEA-2 hr FN/BMP2-coated D900, with ruptured bacterial cells also observed on the nanowires (arrowheads). In the control uncoated samples, large areas of confluent biofilms were observed in contrast to small, more diffuse bacterial accumulation on the nanowire-coated samples. Modified and reproduced with permission from [234].

Important factors in antibacterial efficacy of biomimetic nanotopography include the spacing between two adjacent structures, as well as the aspect ratio. It has been shown that the region of the cell membrane stretched between two neighbouring nanopillars is the critical region that leads to rupture [229]. A recent systematic review summated the common design parameters for nanopattern topographies that result in effective bactericidal activity, based on previous research [230]. The review analysed recent studies of nanopattern design and characterized the optimum height, diameter and interspacing required for achieving antibacterial effect for a plethora of nanopatterns (nanopillars, nanocones, spikes/spinules, nanowires, nanopores, nanopits, nanogrooves). One of the main drawbacks of nanoscale structures and geometries with bactericidal properties (once described as “mechano-bactericidal nanostructures” [231]) is that they can only inactivate bacteria that make direct contact with the nanostructures. Ivanova et al. [232] reported the first successful bactericidal activity of a hydrophilic surface. Modelled on the dragonfly *Diplacodes bipuncata* wing, which naturally possesses high aspect ratio nanoprotrusions, they investigated black silicon using ion etching of silicon as a potential bactericidal approach. Unlike the cicada wing, which only has activity against Gram-negative pathogens, both the dragonfly and its biomimetic analogous black silicon nanopillars were shown to be effective for both Gram-positive and Gram-negative pathogens. Another problem with spiky nanowire topographies includes potential adverse impacts on osteogenesis and repair [233]. To address this issue, one recent study investigated application of a thin, poly(ethyl acrylate) (PEA) coating to Ti nanowires to enhance bioactivity through organization of fibronectin and delivery of bone morphogenetic protein 2 (BMP2) in a bid to enhance mesenchymal stem cell (MSC) adhesion and osteo-specific activity while simultaneously preventing biofilm formation [234]. The hydrophobic PEA allowed proteins, e.g., fibronectin (FN) to displace interfacial water and interact directly with the PEA surface. This organization of FN and presentation of BMP2 on the PEA nanowire substrates enabled enhanced MSC adhesion and reduction of *P. aeruginosa* biofilm formation (Figure 7).

Surface roughness is known to affect the adhesion force of bacterial cells [235]. It has been challenging to precisely engineer nanoscale sophisticated surface structures using simple and scalable techniques. Recently, one group used a scalable, one-step wet etching technique to generate a multiscale topography on aluminium and its alloys [221]. They found an enhanced reduction in the proliferation of *S. aureus* and *E. coli* cells on the altered alloy surface on account of its roughness at multiple scales. The nanostructures in this study were observed to specifically rupture the cell membrane, while the microstructures offered geographical limitation to bacterial adherence. Another promising non-coating method to mitigate bacterial adherence on implant surfaces involves laser surface treatment. One study investigated laser surface treatment on common orthopaedic metallic metals (various Ti grades and a cobalt–chromium–molybdenum composite) and demonstrated the ability to noticeably reduce adhesion of *S. aureus* [236]. Although it is generally accepted that bacteria prefer to adhere to rougher surfaces, this group created surfaces with a higher Ra value and yet attained a reduction in bacterial attachment, rather than an increase. This is thought to be because of “spiky” surfaces in the treated samples, like the bactericidal mechanism of cicada wings.

Suspended colloids may also act as mechano-bactericidal nanostructures. The most investigated colloids are carbon nanotubes, which can act to severely pierce and damage cell membranes of Gram-positive bacteria; their mechanism has led to the dubbing of nanotubes as “nanodarts” [237]. Aside from carbon nanotubes, another class of bactericidal colloidal suspensions frequently reported includes graphene nanosheets, which exert their antibacterial effects similar to carbon nanotubes using penetration-type mechanisms [222].

### 7.2. Surface Charge

The surface of implants can be modified by functionalization with charged groups. Different types of bacteria exhibit differently charged glycocalyces, whereby Gram-negative bacteria are generally associated with a polyanionic glycocalyx and Gram-positive bacteria with polycationic glycocalyx [218]. Implant surfaces that have been functionalised with charged groups can repel bacteria by electrostatic means or promote interactions with adhesive proteins, thereby inhibiting bacterial adhesion. Negatively charged functional groups such as carboxylate and sulfonate groups have been investigated for this purpose [238]. Carboxylate- and sulfonate-functionalised PMMA polymer coatings demonstrated inhibition of *S. aureus* adhesion on discs in vitro despite the presence of a fibronectin coating, which is known to mediate *S. aureus* adhesion on polymer substrates. The surfaces were also observed to support osteoblast function, although this effect depended on optimisation of the chemical composition of the functionalisation groups [239].

### 7.3. Controlling the Hydrophilic/Hydrophobic Properties at the Biological Interface

Increasing hydrophilicity of the implant surface can minimise bacterial attachment by reducing hydrophobic interactions and by minimising protein adsorption [240]. One of the commonest approaches investigated has been to functionalise the surface with the hydrophilic polymer, PEG or poly(ethylene oxide) (PEO). Its anti-fouling properties stem from its flexible and hydrophilic chains that create a wide exclusion volume which can sterically hinder bacterial adhesion [241]. The molecular weight of the PEG can impact on the efficacy. For example, modification of polyurethane surfaces with a higher MW PEG (3.4 kDa) reduced adhesion of *S. epidermidis* and *E. coli* in media and plasma, while lower MW PEG (1 kDa) was not effective [242]. There have been many reports of surface modification with PEG and PEG copolymers for the purpose of minimising implant-related infections. Coating Ti films (smooth surface) or discs (rough surface) with various PEG copolymers, including PEG-polylysine, reduced colonisation by *S. aureus* [243] and by *S. epidermidis, S. mutans* and *P. aeruginosa* [244]. Other examples include PMAA, dextran, or hyaluronic acid [86,89], while heparin coatings have also been employed to generate hydrophilic surfaces, thereby reducing adhesion of bacteria such as *S. epidermidis* [245]. However, a potential drawback with the use of these antifouling hydrophilic surfaces is the inhibition of mammalian cell adhesion, which is problematic in cases where osteointegration is desirable.

Hydrophilic polymer brush coatings on implants have also been developed and investigated as anti-adhesive surfaces. The brush coating forms a dense steric barrier to bacterial attachment. Examples include PEO brushes and poly-acrylamide brushes. A PEO brush coating on glass and silica surfaces successfully inhibited adhesion of a range of bacterial strains in vitro, including *S. epidermidis*, *S. aureus* and *E. coli*, although the reduction was not statistically significant in the case of some strains of the more hydrophobic *P. aeruginosa* [246]. Another study showed that PEO-PPO co-polymer brush coating of silicone surfaces reduced attachment of *S. aureus* and *S. epidermidis* and slowed biofilm formation [247]. Polyacrylamide brushes were covalently introduced onto silicon wafers and resulted in slower and lower rates of bacterial and yeast attachment, compared to uncoated controls [248]. While Ti implants modified with polyacrylamide brushes and the AMP (Tet-20) demonstrated a reduction in bacterial adhesion. In vivo evaluation in the dorsal pocket of a Sprague–Dawley rat model inoculated with 0.25 mL of 1 × 10^8^ *S. aureus* revealed that CFUs decreased by at least 85% on Tet-20-coated implants compared to peptide-free controls in 10 out of 14 rats at 7-days [249].

Nature has provided inspiration for the design of novel repellent surfaces. One of the most prominent examples includes the lotus leaf, wherein the superhydrophobic properties are reputed to be derived from a combination of hierarchical architecture and a hydrophobic waxy surface [250]. Deposition of nanoscale wax crystals on a microstructured papillae surface gives rise to a very high contact angle (>150°) that enables water to easily roll off the leaf [250]. To recapitulate the repellent properties, surface structuring to control the nanotopography and enable contact angles greater than 120° in the design of superhydrophobic surfaces has been undertaken [251,252]. Various synthetic, micro- or nanostructured superhydrophobic surfaces possessing low surface energies that attempt to control the contact area between surfaces and liquids/cells have been investigated and shown to minimise bacterial attachment. One study reported a surface coating composed of a superhydrophobic xerogel based on fluorinated silica colloids, which significantly reduced adhesion of *S. aureus* and *P. aeruginosa* [251]. Femtosecond laser ablation technology was used to engineer the surface pattern of Ti at the micro and nano level leading to an increase in water contact angle from θ_W_ 73 ± 3° to 166 ± 4°. The creation of a superhydrophobic surface on Ti discs was investigated with bacteria possessing two different morphologies. The adhesion of rod-shaped *P. aeruginosa* was inhibited below minimum detectable levels, although *S. aureus* successfully colonised the modified discs, which was attributed to their smaller size and spherical shape [252].

Advances in developing novel non-wetting surfaces include SLIPS technology. This technology was developed to address some of the limitations (e.g., failure under pressure and physical damage) that had restricted the practical application of other liquid-repellent surfaces. Taking their cue again from nature and the *Nepenthes* pitcher plant, researchers designed the SLIPSs technology (slippery liquid-infused porous surface(s)) that was composed of a micro/nanoporous substrate, infiltrated with a lubricating fluid such as liquid perfluorocarbons that prevented adhesion through formation of an immobilised, molecularly smooth, liquid overlayer [253,254]. SLIPS were evaluated for their ability to prevent biofilm formation and adhesion of bacteria, including *P. aeruginosa*. *E. coli* and *S. aureus*, and were found to be vastly superior to alternative superhydrophobic surfaces and to PEGylated surfaces, highlighting their potential for application in the development of self-cleaning and antifouling materials [255]. Extension of this SLIPS technology to create surfaces with antithrombogenic and antifouling potential was undertaken by covalently binding a flexible molecular perfluorocarbon layer on a range of medical-grade materials and then coating it with a mobile layer of perfluorodecalin, a liquid perfluorocarbon. The authors demonstrated an eightfold reduction in biofilm formation on medical grade PVC tubing for up to 6.5 weeks [254]. Another example of a slippery surface focused on BMA-EDMA (poly(butyl methacrylate-co-ethylene dimethacrylate) coated onto glass substrates, when challenged with various strains of *P. aeruginosa*, prevented biofilm formation by some, but not all, of the bacterial strains [256].

**Figure 8:**
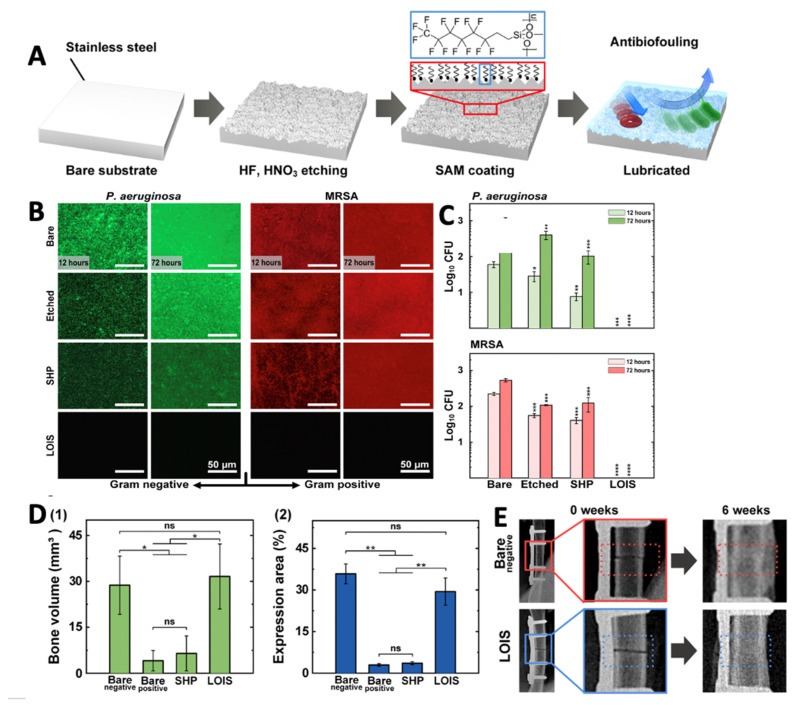
(**A**) Schematic of the fabrication process of LOIS. A stainless-steel bare substrate is chemically etched using hydrofluoric acid (HF) and passivated with nitric acid (HNO_3_) to slow corrosion. The surface is then modified with a self-assembled monolayer (SAM) to increase the chemical affinity between the surface and the subsequently added slippery perfluorocarbon-based lubricant. (**B**) Fluorescence microscopy images of each material (bare, etched, superhydrophobic (SHP), lubricated orthopaedic implant surface (LOIS) incubated in *P. aeruginosa* and *MRSA* suspension for 12 and 72 h. (**C**) Quantification of adherent CFUs of *P. aeruginosa* and methicillin-resistant *Staphylococcus aureus* (MRSA) on each group of surfaces. (**D**) Quantitative analysis of the callus formation outside cortical bone with (1) micro-CT and (2) osteoclast activity based on TRAP activity. (**E**) X-ray images of fractured bone of bare negative (without being exposed to bacterial suspension) surface and LOIS 6 weeks post-implantation. Statistical significance, ns (not significant), * *p* < 0.05; ** *p* < 0.01, *** *p* < 0.001, and **** *p* < 0.0001. Modified and reproduced from [259] under CC BY-NC 4.0 https://creativecommons.org/licenses/by-nc/4.0/ (accessed on 27 July 2022).

Further SLIPS research has sought to understand how modified surface compositions and textures can influence biofilm formation and attachment. Bruchmann et al. assessed *P. aeruginosa* biofilm formation on various patterned SLIPS substrates (superhydrophobic in nature). They found that SLIPS substrates patterned with hydrophilic regions are not resistant to biofilm formation and suggested that biofilms can form provided there are sufficient anchorage points available [257]. Thus, establishing a SLIPS substrate that is completely hydrophobic may be necessary to eliminate biofilm initiation and growth. A variety of structures have been used to hold the lubricant in SLIPS technology, including porous Teflon and epoxy resin and porous polymer layers. One group took advantage of fluoropor, a highly fluorinated polymer foam with adjustable porosity and surface roughness [258]. They showed that surface roughness has an important influence in formation and attachment of a *P. aeruginosa* biofilm, with lower surface roughness leading to reduced biofilm formation. Another group developed a lubricated orthopaedic implant surface (LOIS), a micro/nanostructured implant surface functionalized with fluorine with liquid-repellent and anti-biofouling properties [259]. The authors aimed to mimic the surface of the pitcher plant and thus combined a lubricant layer within the micro/nanostructure of the surface. The lubricant-infused surface minimized contact between biological substances and the surface, enabling anti-biofouling properties. The surface demonstrated virtually no adhesion of *P. aeruginosa* or *MRSA* (Figure 8). Despite many articles demonstrating repellence of such slippery surface materials, this was the first to demonstrate healing of damaged tissue after its implantation. In their rabbit femur fracture model of implant-associated *MRSA* infection, callus formation was greatest in the LOIS group, with similar healing to the bare implant surface (without bacterial exposure) after 6 weeks. Importantly, from a translational perspective, in a surgical simulation ex vivo, the group confirmed the mechanical durability of the implant coating technology against scratches and fixation force.

**Table 4:** Surface feature, drug-free engineering approaches to impede microbial infection in orthopaedic implants. The table highlights surface features, fabrication method and antimicrobial outcome.

Material	Surface Features	Fabrication Method	Bacteria Studied	Antimicrobial Outcome	Reference
Aluminium	Micro- and nano-roughed	Wet etching	*E. coli, K. pneumoniae, P. aeruginosa*	Decreased cell attachment compared to non-etched controls	[221]
Black silicon	High aspect ratio nanoprotrusions 500 nm height; contact angle 80°	Ion etching	*P. aeruginosa, S. aureus, B. subtilis*	Bactericidal; reduces cell viability compared to non-etched controls	[232]
Ti	Nano-roughened; contact angle 59.3 ± 1.13°	Electron beam evaporation	*S. aureus, S. epidermidis, P. aeruginosa*	Decreased adhesion of bacterial colonies compare to conventional, nanotubular and nanotextured Ti	[220]
Ti	Functionalization with PMMA and silk sericin	Atom transfer radical polymerization	*S. aureus, S. epidermidis*	Threefold decrease in number of viable *S. aureus* cells compared to pristine Ti	[260]
Ti	Coating with PEG-polylysine	Polymer surface adsorption	*S. aureus*	Decreased the adhesion of *S. aureus* to the surfaces by 89–93% compared to bare TiO_2_ surface	[243]
Ti	Two-tier micro- and nanoscale surface structures: First tier, large grain-like convex features 10–20 µm in size. Second, ≤200 nm wide irregular undulations on the surface of these grains; superhydrophobic, contact angle 166 ± 4°	Femtosecond laser ablation	*P. aeruginosa, S. aureus*	*S. aureus* colonized the surface. No *P. aeruginosa* cells were able to attach to the surface (i.e., any attached bacterial cells were below the estimated lower detection limit)	[252]
Ti	Micro/nanoscale surface roughness	Etching and adding perfluoropolyether lubricants	*P. aeruginosa*, MRSA	Reduction in log CFU count of *P. aeruginosa* and MRSA to non-measurable	[259]
Ti	Nanopatterned arrays	Hydrothermal etching	*P. aeruginosa,* *S. aureus*	Killed 50% of *P. aeruginosa* cells and about 20% of the *S. aureus* cells contacting the surface	[261]
Ti	Nanocolumnar thin Ti films	Glancing angle sputter deposition	*E. coli, S. aureus*	*E. coli* viability significantly decreased; *S. aureus* viability relatively unchanged	[262]
Ti	Titania nanowire arrays of 100 nm diameter	Alkaline hydrothermal processing	*P. aeruginosa,* *S. aureus*	Selectively bactericidal against *P. aeruginosa* (highly mobile), but not against *S. aureus*	[263]
Ti and CoCrMo alloys	Surface roughened, “spiky” protrusions produced	Continuous wave fibre laser with near-infrared wavelength	*S. aureus biofilm*	Laser treatment of Ti surfaces decreased viable bacteria and biofilm area but effects not evident in laser treated CoCrMo.	[236]
Ti plasma spray implant	Ti nano-spikes	Glancing angle magnetron sputter deposition	*E. coli, S. aureus*	Partial destruction of *E. coli* adherent to the nano-spikes via a physico-mechanical mechanism, not useful against Gram-positive bacteria	[264]
Ti	Nanostructures with peaks and valleys on surface	Etching	*P. aeruginosa, S. aureus*	Decrease in viability of *P. aeruginosa* and *S. aureus* to ~4% and ~40% on nanostructured surfaces, respectively, while viability did not drop below 90% for control surface	[265]

*B. subtilis* (*Bacillus subtilis*), CFU (colony-forming unit), CoCrMo (cobalt–chromium–molybdenum alloy), *E. coli* (*Escherichia coli*), *K. pneumoniae* (*Klebsiella pneumoniae*), *P. aeruginosa* (*Pseudomonas aeruginosa*), PMMA ((poly(methacrylic acid)), *S. aureus* (*Staphylococcus aureus*), *S. epidermidis* (*Staphylococcus epidermidis*), Ti (titanium), TiO_2_ (titanium dioxide).

## 8. Conclusions and Future Perspectives

Orthopaedic medical devices play a crucial role in restoring functionality to patients suffering from debilitating conditions, but their surgical implantation carries a risk of infection, with a profound impact for patients and healthcare providers [266]. Given the increasing patient life expectancy and anticipated increase in the number of orthopaedic implantation procedures that will be undertaken, orthopaedic device infection represents a salient problem for all stakeholders. This problem has culminated in a concerted research focus and a broad spectrum of approaches and technology solutions being proposed to address this problem. Despite research efforts, there has been little translation of novel, effective solutions to the clinic.

Various approaches (passive and active) to minimise bacterial contamination of implants and the development of infection have been investigated. The prospect of locally delivering antimicrobial agents from the implant surface addresses many of the limitations of systemic administration, e.g., poor perfusion of drug to the implant site in sufficient therapeutic concentrations. Local targeting overcomes the necessity for high dose, systemic administration and offers the prospect for controlled drug delivery. Despite these advantages, concern exists in relation to the risk of drug resistance with routine use and whether therapeutic drug concentrations over the desired timeframe are achievable. It is possible that coatings can prevent infection in the acute phase, but there is a paucity of data regarding their performance in chronic infections [267]. Prolonged antimicrobial delivery proximal to the implant site usually requires a platform technology to control the presentation of the antimicrobial cargo. Biomaterials including polymers and ceramics have been intensively investigated for this purpose, but problems with biocompatibility, foreign body reaction and device performance may complicate technology development. Coating orthopaedic devices with a single antimicrobial agent is also limited by differences in the causative organism and the susceptibility to treatment, and an increasing prevalence of polymicrobial and resistant infections. Despite these limitations, evidence has shown that antibiotic coated implants are beneficial in the management of high-risk patients with open fractures and the higher implant costs can be offset by the savings due to lower infections, reduced hospital stays and procedures [170].

There is also interest in developing alternative strategies to those involving traditional antimicrobial drugs. This stems primarily from the increasing challenge of drug resistance and the lack of new antimicrobial drugs being developed. Examples of new therapeutic moieties include AMPs, nanomaterials with intrinsic antimicrobial activity and agents that interfere with quorum sensing and biofilm formation. At the same time, research has increasingly focused on engineering device features to prevent bacterial attachment and biofilm formation. Drug-free strategies also offer the advantage of simplifying the regulatory pathway compared to drug-device combination products. Multi-prong, synergistic strategies that work at multiple targets against infection or approaches that differentially engineer surfaces to confer osteogenic potential, while also inhibiting infection are increasingly being researched and offer much promise [95]. However, many of these novel technologies and approaches are at an earlier stage in their development and require further investigation. Further studies are required to address challenges including toxicological concerns associated with the use of nanotechnology [202], and further exploration of the potential microbial resistance to nanomaterials and nano-engineered surfaces is warranted over timeframes that approximate their usage [268]. Studies have been predominantly conducted in vitro, with fewer in vivo studies [201]. Given that a disconnect between performance in vitro and in vivo can exist, it is important to conduct in vivo studies and ensure that models and procedures mimic the infection scenario [269,270]. To this end, there is also a need to develop robust, standardised, quantitative, and cost-effective methodologies both in vitro and in vivo that accurately model physiological environments and include established biofilm models to provide sound evidence of safety and efficacy and ensure the most promising technologies are translated. Relatedly, the development of standardized guidelines appropriate to assess the biocompatibility and toxicology of novel technologies would enable a more timely and accurate assessment of potential technologies. The translation of next-generation devices that minimise device related infection has also been impacted by a global divergence in the regulatory pathway requirements in different markets and ongoing changes in the regulatory requirements for medical devices, especially in the case of combination devices.

Furthermore, it is important to ensure that novel solutions readily translate to the manufacture setting, do not act as a barrier to scale-up or adversely impact on surgical implantation, or significantly add to the implant cost. Considering the significant challenges posed by orthopaedic device-related infection and the promising array of technologies to address this problem, a coordinated and proactive approach between all the major stakeholders (researchers, industry, regulators, reimbursement, and patient groups) is required to ensure that patients can safely access transformative and cost-effective technologies.

## Figures and Tables

**Table 1:** Considerations in the design of orthopaedic devices to mitigate device-related infections.

Property	Features
Infection	Bacteria species, strain, properties (charge), susceptibility to treatment, presence of biofilm
Device	Material (metal–titanium alloy, stainless steel; polymer—PEEK), Shape Application—arthroplasty, fracture fixation, trauma Permanent or temporary implantation
Infection control strategy	Antimicrobial surfaces (antimicrobial release or contact inhibition) Anti-adhesive surfaces, bacteria repellent. Interference with biofilm formation (e.g., quorum-sensing inhibitors, quorum quenchers, enzymes, small molecules, immunotherapy)
Antimicrobial cargo	Controlled release profile—spatiotemporal control over presentation of antimicrobial agents, reproducible PK/PD parameters Properties—MIC, pathogen selectivity, spectrum of activity, species selectivity, mode of action, toxicity to host cells and tissue, resistance
Surface properties	Surface roughness, chemistry, energy, and wettability Physical architecture—nanotopography Interaction with host proteins, host cells and bacteria Influence on cell proliferation and differentiation
Mechanical features	Sufficient to facilitate handling and surgical implantation. Replicate those of the target tissue to provide important cues that instruct tissue development where integration is desirable while simultaneously limiting stress shielding
Biomaterial properties	Biocompatibility—material and breakdown products should be biocompatible to avoid foreign body responses that might otherwise lead to rejection or interfere with the healing cascade. Host responses Where integration is desirable, biomaterials should ideally be osteoconductive, osteoinductive and support osseointegration.
Manufacture/ Production	Technology should facilitate translation from bench scale to the clinic. Production according to GMP standards. Cost-effective.
Development and Regulation	Preclinical (in vitro, in vivo) and clinical models. Regulatory requirements—e.g., testing the efficacy of combination medical devices. Antimicrobial efficacy according to ASTM standards Approval pathway and regulatory requirements in different jurisdictions.

GMP (good manufacturing practice), MIC (minimum inhibitory concentration), PEEK (polyether-ether-ketone), PK/PD (pharmacokinetic/pharmacodynamic).

## Data Availability

Not applicable.
